# Electron population dynamics in resonant non-linear x-ray absorption in nickel at a free-electron laser

**DOI:** 10.1063/4.0000206

**Published:** 2023-10-11

**Authors:** Robin Y. Engel, Oliver Alexander, Kaan Atak, Uwe Bovensiepen, Jens Buck, Robert Carley, Michele Cascella, Valentin Chardonnet, Gheorghe Sorin Chiuzbaian, Christian David, Florian Döring, Andrea Eschenlohr, Natalia Gerasimova, Frank de Groot, Loïc Le Guyader, Oliver S. Humphries, Manuel Izquierdo, Emmanuelle Jal, Adam Kubec, Tim Laarmann, Charles-Henri Lambert, Jan Lüning, Jonathan P. Marangos, Laurent Mercadier, Giuseppe Mercurio, Piter S. Miedema, Katharina Ollefs, Bastian Pfau, Benedikt Rösner, Kai Rossnagel, Nico Rothenbach, Andreas Scherz, Justine Schlappa, Markus Scholz, Jan O. Schunck, Kiana Setoodehnia, Christian Stamm, Simone Techert, Sam M. Vinko, Heiko Wende, Alexander A. Yaroslavtsev, Zhong Yin, Martin Beye

**Affiliations:** 1 Deutsches Elektronen-Synchrotron DESY, Germany; 2 Department of Physics, Universität Hamburg, Hamburg, Germany; 3 Department of Physics, Imperial College London, London, United Kingdom; 4 Faculty of Physics and Center for Nanointegration Duisburg-Essen CENIDE, University of Duisburg-Essen, Duisburg, Germany; 5 Institute for Solid State Physics, The University of Tokyo, Kashiwa, Chiba 277-8581, Japan; 6 Ruprecht Haensel Laboratory, Deutsches Elektronen-Synchrotron DESY, Germany; 7 Institut für Experimentelle und Angewandte Physik, Christian-Albrechts-Universität zu Kiel, Kiel, Germany; 8 European XFEL, Schenefeld, Germany; 9 MAX IV Laboratory, Lund University, Lund, Sweden; 10 Sorbonne Université, CNRS, Laboratoire de Chimie Physique-Matière et Rayonnement LCPMR, Paris, France; 11 Paul Scherrer Institut, Villigen, Switzerland; 12 Debye Institute for Nanomaterials Science, Inorganic Chemistry and Catalysis, Utrecht University, Utrecht, The Netherlands; 13 The Hamburg Centre for Ultrafast Imaging CUI, Hamburg, Germany; 14 Department of Materials, ETH Zurich, Zurich, Switzerland; 15 Helmholtz-Zentrum Berlin für Materialien und Energie GmbH, Berlin, Germany; 16 Max Born Institute for Nonlinear Optics and Short Pulse Spectroscopy, Berlin, Germany; 17 Institute for Electric Power Systems, University of Applied Sciences and Arts Northwestern Switzerland, Windisch, Switzerland; 18 Institute for X-ray Physics, Göttingen University, Göttingen, Germany; 19 Department of Physics, Clarendon Laboratory, University of Oxford, Oxford, United Kingdom; 20 Central Laser Facility, STFC Rutherford Appleton Laboratory, Didcot, United Kingdom; 21 Department of Physics and Astronomy, Uppsala University, Uppsala, Sweden; 22 International Center for Synchrotron Radiation Innovation Smart, Tohoku University, Sendai, Japan; 23 Laboratorium für Physikalische Chemie, ETH Zürich, Zürich, Switzerland

## Abstract

Free-electron lasers provide bright, ultrashort, and monochromatic x-ray pulses, enabling novel spectroscopic measurements not only with femtosecond temporal resolution: The high fluence of their x-ray pulses can also easily enter the regime of the non-linear x-ray–matter interaction. Entering this regime necessitates a rigorous analysis and reliable prediction of the relevant non-linear processes for future experiment designs. Here, we show non-linear changes in the $L_{3}$-edge absorption of metallic nickel thin films, measured with fluences up to 60 J/cm^2^. We present a simple but predictive rate model that quantitatively describes spectral changes based on the evolution of electronic populations within the pulse duration. Despite its simplicity, the model reaches good agreement with experimental results over more than three orders of magnitude in fluence, while providing a straightforward understanding of the interplay of physical processes driving the non-linear changes. Our findings provide important insights for the design and evaluation of future high-fluence free-electron laser experiments and contribute to the understanding of non-linear electron dynamics in x-ray absorption processes in solids at the femtosecond timescale.

## I. INTRODUCTION

The modern understanding of complex materials relies on suitable approximations to the unabridged quantum mechanical description of the full, correlated many-body problem. To assess the predictive power of theoretical models and the selected approximations, detailed experimental studies of systems driven out of equilibrium are particularly insightful.

X-ray free-electron lasers (FELs) with their high-power (about 10^10^ photons/pulse after monochromatization) and ultrashort pulses (tens of fs) are uniquely suited for probing the non-equilibrium dynamics of matter in all states from solid state to plasma.^1,2^

While schemes with separate pump and probe pulses can be used to observe dynamics slower than the pulse durations, transmission measurements of individual intense pulses can be used to infer the x-ray interaction and material dynamics within the pulse duration. With increasing pulse energy, the interaction with an exposed solid becomes highly non-linear, as the material state changes during the interaction. In non-linear absorption measurements, a single intense x-ray pulse acts as both a pump and a probe at the same time, and its total absorption depends on the changes it induces in the electronic system.^3–9^ The highly excited solid evolves toward a state of warm dense matter (WDM) where individual electronic excitations reach up to hundreds of eV and excitation energies average out to many eV per atom, while the nuclear lattice still resembles that of the cold solid during the femtosecond pulse.^10–19^

In this paper, we present new fluence-dependent x-ray absorption spectra recorded with monochromatic x rays on metallic nickel thin films around the nickel 2 $p_{3/2}$ ( $L_{3}$) edge, revealing changes in the valence electron system around the Fermi level, driven by FEL excitation densities spanning several orders of magnitude from the linear regime up to 60 J/cm^2^ (corresponding to 2 $\times 10^{15}$ W/cm^2^).

The electronic processes that ensue after the absorption of core-resonant photons trigger a complex series of dynamical processes of photon-absorption, electron excitation, and subsequent electronic scattering. Such non-equilibrium processes with a large amount of correlated particles involved and spanning orders of magnitude in internal energy are challenging to treat in *ab initio* simulations.^20–24^ Rather than striving for a direct time-dependent solution to the quantum-mechanical many-body problem, our analysis, therefore, explores how much of the observed effects can be explained purely by the evolution of electronic populations within the ground-state density of states (DOS) and scaling the known ground-state probabilities of absorption, decay, and scattering processes with the current populations of the participating states. Therefore, we develop a rate equation model to provide an intuitive understanding of the electronic processes. Our model successfully describes the largest part of the non-linear changes in the spectra, affirming the dominant role of electron redistribution. However, especially in close vicinity of the resonance, our measurements deviate from the predictions of the rate model and indicate the need for more sophisticated theories. Nevertheless, our straightforward picture of an intense core-resonant x-ray pulse interaction with the valence system of a 3d metal lays a solid knowledge-based foundation for the planning and interpretation of non-linear x-ray spectroscopy experiments at FELs.

This paper is structured as follows: Sec. II describes the experimental setup used to acquire non-linear XANES spectra of the L_3_-edge of metallic nickel films. In Sec. III, we introduce the working principle of the rate model used to interpret the experimental data. In Sec. IV, we show the experimental data in direct comparison to the simulation results and continue to interpret our findings in Sec. V. We conclude in Sec. VI.

## II. EXPERIMENT

X-ray absorption spectra of the nickel 2 $p_{3/2}$ ( $L_{3}$) edge were recorded at the Spectroscopy and Coherent Scattering Instrument (SCS) of the European XFEL.^25^

**FIG. 1.**
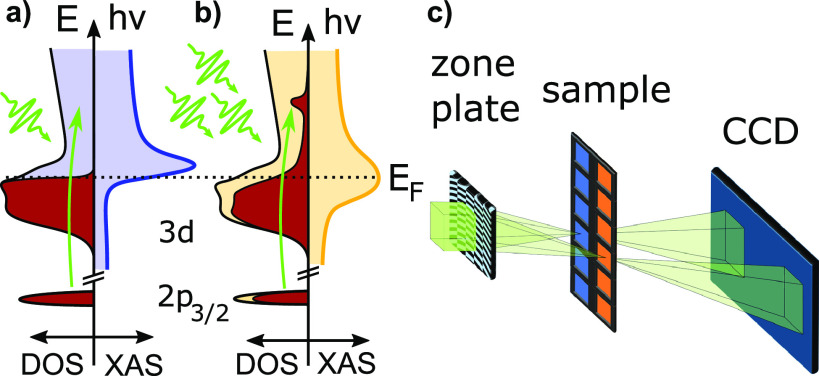
(a) Absorption at low fluences. The electronic system remains mostly in the ground state. The left side shows the density of states, red occupied and blue unoccupied, while the right side displays the resulting spectrum. (b) Absorption at high fluence. Later parts of the x-ray pulse probe a hot electronic system and see less unoccupied valence states at the resonant energy (bleaching). Unoccupied states and spectrum are shown in yellow. (c) Setup for non-linear XAS. The split-beam-normalization scheme uses a special zone plate,^29^ which generates two adjacent beam foci for transmission through the sample and a reference membrane before the beams impinge on the detector.

The XAS (x-ray absorption spectroscopy) spectra were measured by continuously scanning the SASE3 monochromator^26^ (synchronized with the undulator gap) back and forth many times in the range 846–856 eV. The photon bandwidth was about 420 meV full-width half-maximum (FWHM) and the FEL pulse duration on the sample was about 30 fs FWHM. The polarization was linear horizontal. The overall beam intensity was controlled using a gas attenuator filled with nitrogen and monitored using an x-ray gas-monitor (XGM) downstream of the monochromator.^27,28^ Figure 1 illustrates the experimental concept. Panels (a) and (b) display the DOS (integrated over spin-up and spin-down states) next to the resulting XAS spectrum (including non-resonant background); panel (a) shows the situation of a low-fluence measurement, panel (b) shows the situation where sufficient photons are used to alter the DOS occupation and hence the measured XAS spectrum. Panel (c) shows the geometry of zone-plate, sample, and detector.

For x-ray absorption measurements at FELs based on self-amplified spontaneous emission (SASE), beam-splitting schemes can deliver optimal normalization of SASE-fluctuations.^30–32^ Here, we utilize such a scheme using a focusing and beam-splitting zone plate which also creates the required tight focusing to achieve extreme fluences. The zone plate combines an off-axis Fresnel structure for focusing and a line grating for beam-splitting in a single optical element.^29^ It thus produces two *μ*m-sized, identical foci in the sample plane, 1.9 mm apart, originating from the first-order diffraction of the zone plate, as well as the positive and negative first orders of the line grating. The sample has a square support of 25 mm size, containing square Si_3_N_4_ membrane windows (orange in Fig. 1) of (0.5 × 0.5) mm^2^ size and 200 nm thickness with a distance of 2 mm between adjacent windows. Every second pair of rows (blue in Fig. 1) was additionally coated with a 20 nm thick sample layer of polycrystalline metallic Ni by sputter deposition, on top of a 2 nm bonding layer of Ta; a 2 nm Pt capping layer was applied to prevent oxidation during sample-handling.

The sample frame was positioned such that one zone plate focus impinged on a nickel-coated membrane, while the other hit a bare silicon-nitride membrane. Thus, the difference in transmission of both beams can be dominantly attributed to absorption in the nickel film.

The detector was a fast readout-speed charge-coupled device (FastCCD) with a high dynamic range, enabling 10 Hz readout and increasing the fluence range available to the experiment.^33–35^ Due to an unstable detector temperature, significant retroactive calibration of the detector was necessary (see Subsection 2 of Appendix B). To prevent detector saturation during measurements with the unattenuated beam, an additional aluminum filter of about 13 μm thickness was used between sample and detector.

During these high-intensity measurements, sample and reference films were locally damaged by intense individual FEL shots. Thus, the FEL was operated in single-shot mode at 10 Hz repetition rate, and the sample was scanned through the beam continuously at 0.5 $\text{mm}\;s^{-1}$, resulting in ten shots per membrane window.

The shot craters in the reference membranes were later analyzed with scanning electron microscopy (SEM) to determine the effective focal size at specific photon energies. The resulting spot sizes were used to calibrate ray-tracing calculations which delivered the photon-energy-dependent spot size, ranging from 0.4 $\mu m^{2}$ to about 3 $\mu m^{2}$ (see Subsection 4 of Appendix B for details on the spot size determination).

## III. MODELING

Various approaches have been proposed to describe the interplay between photon absorption and the electronic structure evolution during the absorption of an FEL pulse. *Ab initio* methods such as Monte Carlo calculations, which explicitly calculate a large number of individual particles' interaction pathways,^36^ and time-dependent density functional theory (TD-DFT), which sets out to solve the full quantum-mechanical many-body problem in terms of the electron density,^37^ generally scale poorly with particle number. In contrast, rate models provide a simpler yet useful tool by describing the interplay between photon absorption and electronic system using non-quantized volume-average quantities and rates directly on a macroscopic scale.^5,38–41^

Away from material resonances, rate models have been successfully used to describe fluence-dependent x-ray absorption in three-level systems, representing the ground, core-excited, and intermediate valence-excited states.^5,38^ When probing the valence bands around material resonances, however, the evolution of the electronic system requires explicit modeling of the energy-resolved valence state populations.^42^ Tracking the full non-thermal population history proved crucial for accurately describing the non-linear absorption changes near and around the Fermi level. We assume that on the modeled femtosecond timescale, the DOS does not change significantly, which is motivated by the slower lattice reaction. This approach though cannot capture subtle changes in electron correlations.^43^ Furthermore, since our measurements are done with linear polarization and are therefore not sensitive to the magnetization, we do not consider the exchange-split DOS but rather integrate over minority and majority electrons.

We describe the propagation of x-ray photons through the sample as well as the dynamics of electron populations within the sample using a set of ordinary differential equations. These are assembled from terms that each describe the rate of a specific physical process. The rate of each process is based on a tabulated or measured ground-state parameter, such as the Auger-lifetime or the absorption cross section, scaled with the appropriate fractional occupations at the simulated time.^44^ The relevant process rates are compiled into differentials of electronic populations and photon density in space and time and implemented in a finite-element simulation to derive the electron population history and ultimately the x-ray transmission of a three-dimensional sample. Only the time constants for the valence band thermalization and the scattering cascades of free electrons are treated as free parameters and adapted to fit the experimental data.

The model considers an idealized three-dimensional sample traversed by an x-ray pulse with Gaussian shape in space and time. We make key approximations to reduce computational effort, such as neglecting any movement of electrons within the sample [consider the inelastic mean free path of relevant photoelectrons of about 1.3 nm (Ref. 45)] and describing photon propagation exclusively in the forward direction. The temporal evolution is solved using the fourth-order Runge–Kutta method with adaptive time-stepping. The propagation of photons in space is calculated as if it happened instantaneously in between the time-steps using the explicit fourth-order Runge–Kutta method.

In order to account for the two-dimensional Gaussian transversal intensity profile of the FEL spot, we first calculate the transmission of the sample for transversally uniform illumination for different fluences. Since we omit transversal coupling, the response to the Gaussian beam profile can then be reconstructed by appropriate radial integration over many values obtained for constant illumination. With these simplifications, the overall computational complexity is drastically reduced, as we simplify a problem with partial differentials in four dimensions into two separable one-dimensional initial value problems, one for photon propagation in space and one for the evolution of electronic populations in time.

**FIG. 2.**
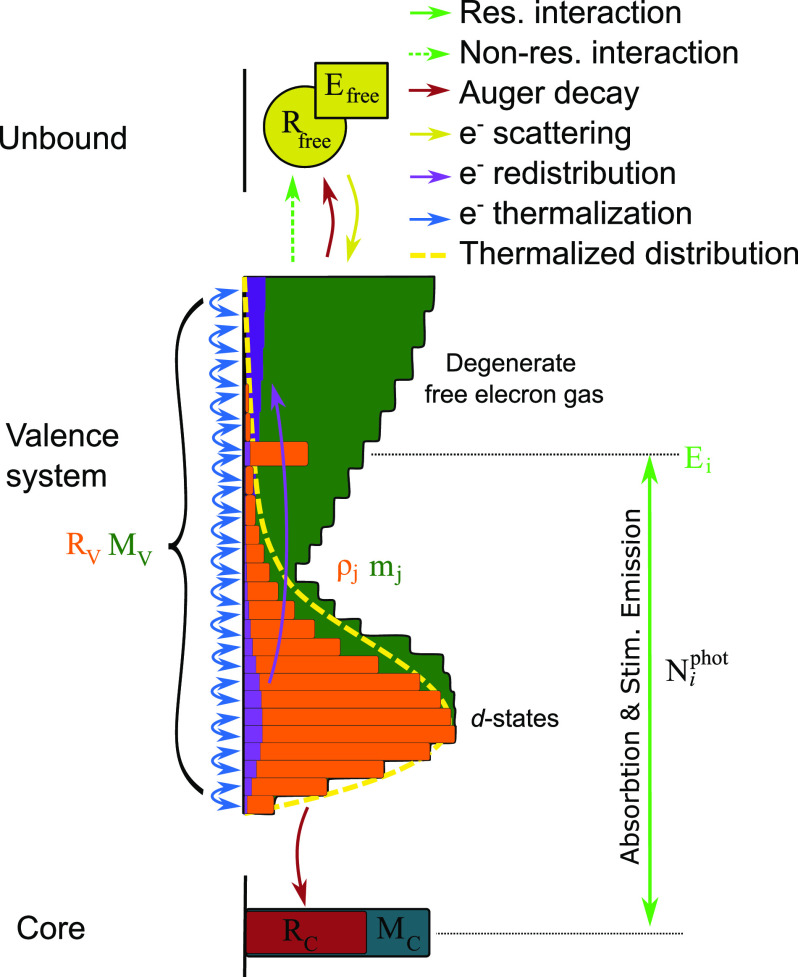
Photon, electron, and energy densities and their interactions. A photon density $N_{i}^{\text{phot}}$ drives resonant interactions between the core electrons *R_C_* and specific valence electrons *ρ_j_*. It also drives non-resonant excitations from the entire valence electron system $R_{V}=\sum_{j}\rho_{j}$ to free electrons *R*_free_, which have a total energy of *E_free_*. Auger–Meitner decays transfer electrons from the valence system to both core states and free electrons; scattering cascades transfer electrons and energy from the free states to the valence system; thermalization drives the valence system toward a thermalized Fermi–Dirac distribution. $M_{C},M_{V}$, and *m_j_* represent the number of available states and are pictured as bars to represent the energy bins of the numerical calculation.

The model describes the interaction between three types of population densities of electrons as well as incident photons via six distinct physical processes, listed in Tables I and II, respectively. Figure 2 schematically illustrates their relationships. The electron populations *R_C_* and *R_V_* describe the total number of electrons bound in the core and valence system, respectively, for an average single atom in the sample. Their values are limited by the number of available states, *M_C_* and *M_V._* In the presented nickel L_3_-edge spectra, the ground-state populations are *R_C_* = 4, representing the 2 $p_{3/2}$-electrons and *R_V_* = 10, representing electrons from the 3d and 4s states. We describe the electronic population of the valence system in an energy-resolved manner, splitting it up into a discrete number of densities *ρ_j_*, where *j* represents the index along the valence energy axis. The number of available states *m_j_* for each energy bin in the valence system is derived from the calculated ground-state DOS^46,47^ up to 30 eV above the Fermi level *E_F_*. Beyond this value up to 800 eV above the Fermi level, the DOS of a free electron gas is used.^22^ All electrons with even higher energies, such as photo-electrons created via non-resonant absorption and Auger-electrons from the decay of core-holes, are described in a separate pool of electrons *R_free_* without energy resolution, although the total energy of electrons in this pool is tracked by the parameter *E_free_*.

**TABLE I.** Electron and photon numbers in the rate model.

*R_C_*	Number of 2 $p_{3/2}$ ( $L_{3}$) core electrons, populating the available core states *M_C_*
*R_V_*	Number of valence electrons, populating available valence states *M_V_*; subdivided by energy bins with *ρ_j_* and *m_j_*, which describe the energy-resolved DOS and its population
*R*_free_	Number of free electrons, created by Auger decay or photoemission before scattering with valence electrons
$N_{i}^{\text{phot}}$	Density of x-ray photons incident per area at photon energy *E_i_* in one simulation time step

**TABLE II.** Process rates in the simulation: The index *j* enumerates the specific energy level *E_j_* within the valence system; *i* enumerates the incident photon energy *E_i_.*

$P_{j}^{\text{res}}$	Rate of resonant interaction, i.e., absorption and stimulated emission between core level and bound valence states
$P_{i,j}^{\text{non}-\text{res}}$	Rate of non-resonant absorption from bound valence states to the unbound free electrons
$P_{j}^{\text{Auger}}$	Rate of Auger decay from the valence system to the core level
$P^{\text{scatt}}$	Rate of electron scattering of unbound electrons returning to the valence system
$P_{j}^{\text{red}}$	Redistribution of valence electrons due to cascading $P^{\text{scatt}}$ electrons
$P_{j}^{\text{therm}}$	Rate of thermalization of the valence system toward a Fermi–Dirac distribution

The processes of resonant absorption and stimulated emission are summarized in the resonant interaction process $P_{j}^{\text{res}}$. This is possible because both processes describe a transfer of electrons between the 2 $p_{3/2}$ core level and the valence system following the same interaction cross section, just with inverted sign. The non-resonant absorption process $P_{i,j}^{\text{non}-\text{res}}$ describes photon absorption at all other electronic levels. The non-resonant absorption length of the ground state is derived from the measured pre-edge absorption. Because of the relatively small contribution of the intermediate 3*p* and 3*s* electrons, the model ascribes all non-resonant absorption events to valence electrons, transferring them to the pool of high-energy electrons *R_free_*. Sequential two-photon absorption (TPA) processes are implicitly treated by the model as a resonant absorption event followed by a non-resonant absorption event. This description does not account for the coherence and resonant enhancement of the TPA process. However, the fluences used here do not exceed 1.5 $\times 10^{31}\text{photons}{\text{cm}}^{-2}\;s^{-1}$, where according to the scaling law proposed by Szlachetko *et al.*,^48^ the relative contribution of the direct TPA process should be on the order of 1%. Thus, the inaccuracy caused by not treating this process explicitly should be very small. Apart from photoelectrons from the valence system, the Auger–Meitner decay process $P_{j}^{\text{Auger}}$ likewise contributes to the free electron pool. These free electrons proceed to scatter with the valence electron system. The total rate of scattering $P^{\text{scatt}}$ is modeled by a simple lifetime parameter $\tau_{\text{scatt}}$, which characterizes how quickly free electrons re-join the valence system. The redistribution process $P_{j}^{\text{red}}$ distributes the kinetic energy of each scattering free electron among the valence system. Avoiding the complexity of explicitly calculating the multiple collisions involved in these electron scattering cascades, the algorithm instead approximates the effect of such a cascade: The energy of the scattering electron is spent to elevate an evenly distributed fraction of all valence electrons from occupied states to locally available unoccupied states (as indicated in purple in Fig. 2). Note that this process is not independent but represents an immediate consequence of the free electron scattering process; the scattering time $\tau_{\text{scatt}}$, thus, characterizes both $P^{\text{scatt}}$ and $P_{j}^{\text{red}}$ together. Finally, electronic thermalization is modeled with a bulk timescale $\tau_{\text{th}}$ (essentially quantifying electron–electron scattering) that moves the non-thermal valence electron distribution toward a target Fermi–Dirac distribution that corresponds to the momentary internal energy and population of the valence system.

The full mathematical description of the process terms is given in Subsection 1 of Appendix A, while the choice and derivation of input parameters is detailed in Subsection 3 of Appendix A.

### A. Differentials

From these process terms we assemble the time-differentials of the populations of electrons and photons listed in Table I. The movement of electrons between states is represented by process terms of electronic transitions appearing symmetrically in these differentials with positive and negative signs, thus ensuring the conservation of particle number. For example, the term for Auger decay appears twice with a negative sign in the valence electron differential, and once each with a positive sign in the differential for core- and free electrons.

The number of photons is reduced or increased by resonant interaction and reduced by non-resonant absorption. The model allows for an arbitrary number of incident photon energies *E_i_*, each of which must be resonant to a specific bin of the valence energy system *E_j_*. The temporal intensity profile of all incident photons is modeled as Gaussian. For the presented calculations, only a single resonant photon energy was used, representing measurements with monochromatic x rays. Incident photons are the only source of energy flow into the system, and all energy eventually emerges as the thermal energy of the valence system.

The valence system interacts via all modeled processes. The resonant absorption rate $P_{j}^{\text{res}}$ changes the valence electron densities *ρ_j_* at all incident photon energies *E_i_* that are resonant. Via non-resonant absorption, incident photons can be absorbed by electrons from all valence states *ρ_j_*. Auger processes depopulate the valence system. The thermalization drives electrons toward the Fermi–Dirac distribution based on the current internal energy and population of the valence band, without changing the total valence occupation. Electron scattering *P*^scatt^ causes electrons from the free electron pool to re-join the valence system in a random unoccupied state *h_j_*, as well as redistributes electrons inside the valence system in an electron cascade triggered by the process, which is described via the $P_{j}^{\text{red}}$ term.

The population of core electrons is reduced (or increased, depending on the sign of $P_{i,j}^{\text{res}}$) by resonant transitions of all incident photon energies *E_i_* to states at all resonant energies *ρ_j_*, and is increased by Auger decay from electrons of all energies *j* in the valence system. Note that the radiative emission channel is neglected in our model as it is designed for soft x-ray energies where Auger emission accounts for most core-hole decays (here specifically, 99.1% of the nickel L_3_ core-hole decays^49,50^). In another concession to the specific experiment simulated here, we further neglect fast electrons leaving the sample, since the electron mean free path of about 1.3 nm (Ref. 45) is much shorter than the sample thickness of 20 nm. While the model is generally suited to implement a loss process for free electrons, the total number of electrons in the system being strictly constant over time is a valuable indicator for the self-consistency of the calculation.

Free electrons are generated by non-resonant absorption from all incident photon energies *E_i_* as well as Auger-decays from all energies in the valence band. The population is reduced by the free electron scattering rate $P^{\text{scatt}}$.

See Subsection 2 of Appendix A for the differential equations.

## IV. RESULTS

**FIG. 3.**
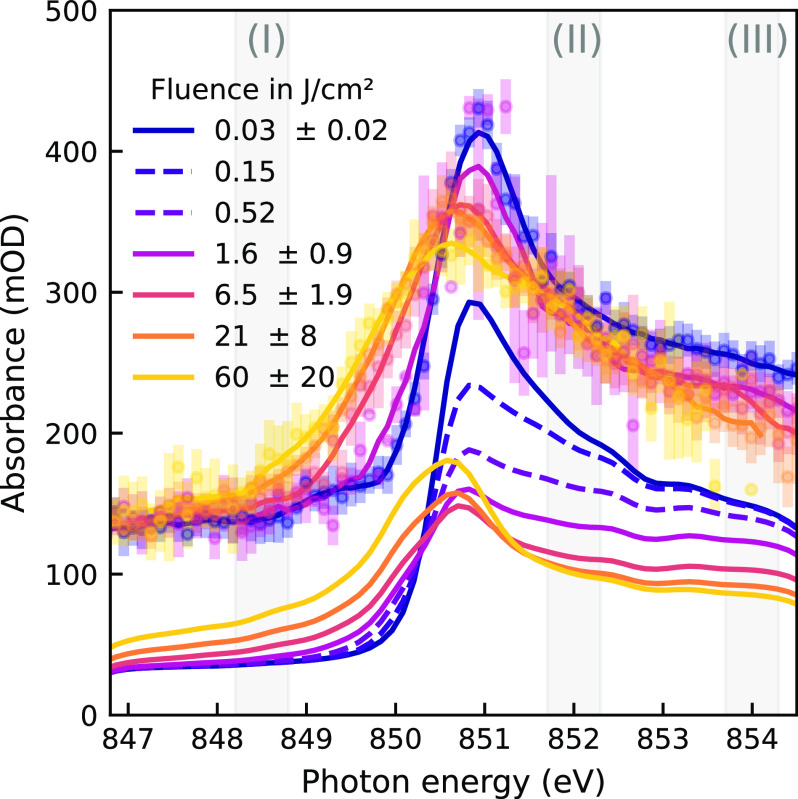
Fluence-dependent Ni $L_{3}$-edge spectra, measured (top) and simulated (bottom). The fluence of events contributing to each spectrum is given in the legend in terms of mean and standard deviation. Dashed simulated spectra do not have a corresponding measurement. The regions of interest from which absorbance changes shown in panels (b), (d), and (e) of Fig. 4 were quantified are shaded and labeled (I) (II), and (III), respectively. The error bars are shown for the measured spectra and represent the 95% confidence intervals for each bin of 102 meV width; solid lines of the measured spectra are smoothed using a Savitzky–Golay filter using windows of 21 bins and fourth-order polynomials. The experimental spectra are vertically offset by 100 mOD.

Figure 3 shows the measured spectra for the nickel $L_{3}$-edge next to simulated spectra for increasing x-ray fluence over more than three orders of magnitude, from 0.03 to 60 J/cm^2^. Each measured point represents an average of several FEL shots, sorted by x-ray fluence and photon energy. The varying statistical uncertainty is a result of the pulse intensity fluctuations of monochromatized SASE radiation^51^ in combination with photon energy-dependent spot sizes (see Subsection 3 of Appendix B for details on the shot classification).

**FIG. 4.**
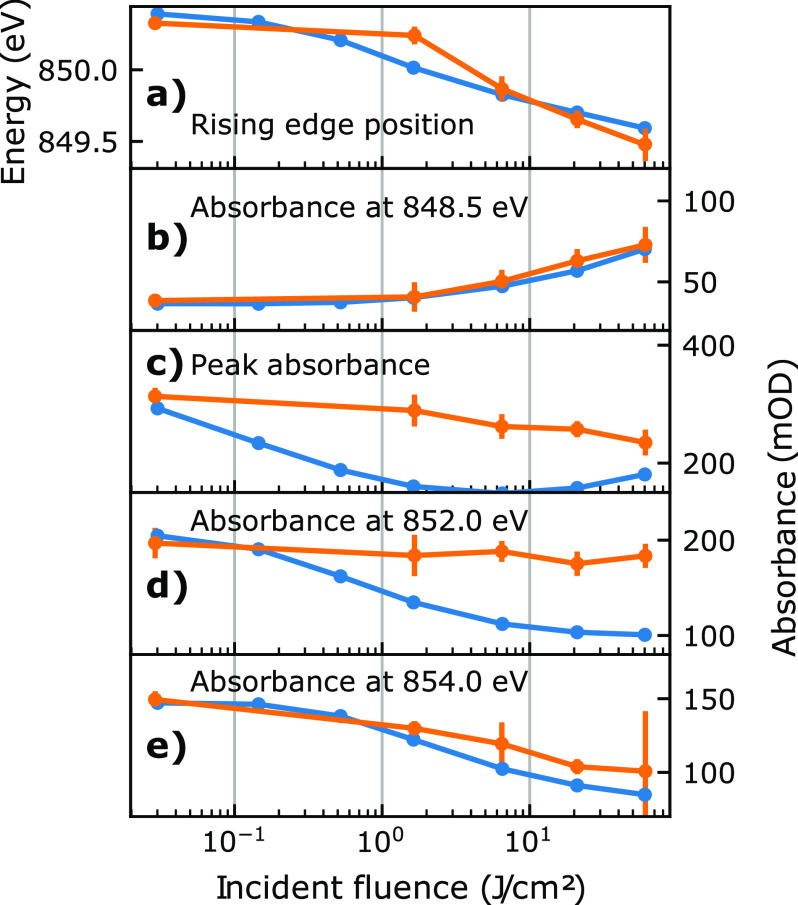
Comparison of spectral effects between simulation (blue lines) and experiment (orange lines with error bars). The shift of the absorption edge in panel (a) represents the photon energy at which the half-maximum of the absorption peak is reached. The absorbance changes in panels (b), (d) and (e) are integrated from the gray shaded regions in Fig. 3, while panel (c) shows the global maximum of the spectrum.

We observe four main fluence-dependent effects, which we quantify and compare to the simulated results in Fig. 4: (a) a redshift of the absorption edge of up to 0.9 ± 0.1 eV in the rising flank; (b) an increase in the pre-edge absorbance, as the rising edge of the absorption peak shifts and broadens; (c) a reduced peak absorbance; and (d) and (e), a reduced post-edge absorbance. The integration regions from which the effects (b), (d), and (e) are derived, are highlighted in Fig. 3 as (I), (II), and (III), respectively. Each is 0.6 eV wide. The shift of the absorption edge is quantified by the photon energy at which the absorbance reaches half of the peak value; its uncertainty is propagated from the statistical uncertainty of the absorption peak measurement.

As apparent from Fig. 4, the rise in absorption at the pre-edge (region I), the drop in post-edge absorption (region III), as well as the shift of the rising edge show good agreement within the measurement uncertainties between simulation and experimental data. The deviations observed in the absorption level of the resonance peak and just beyond will be discussed later.

We emphasize that this level of agreement with the experimental data are achieved across more than three orders of magnitude in fluence, based on a rather simplified description of well-known physical processes in combination with experimental or tabulated ground-state properties such as density, electronic configuration, and ground-state spectrum. Only the valence thermalization time $\tau_{\text{th}}$ and electron scattering time *τ_scatt_* were varied to achieve the best match to the experimental results. We obtain a value of $\tau_{\text{th}}=6$ fs, which compares well to recent estimates for excitations on this energy scale.^40,41,52,53^ The scattering time constant $\tau_{\text{scatt}}=1.5$ fs produces the best agreement with experimental data. This value appears reasonable as it summarizes a cascade of many individual electron scattering events, which we would expect to occur roughly every 100 as.^45^

## V. DISCUSSION

**FIG. 5.**
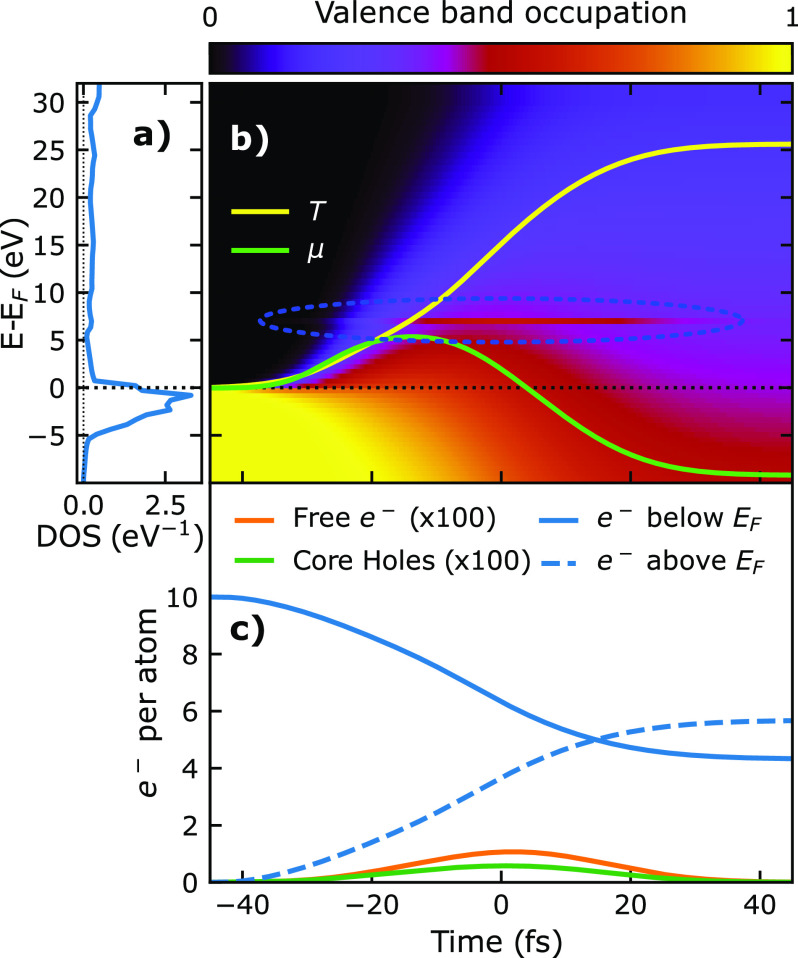
Evolution of electronic populations (simulation) in a single voxel at the sample surface for a pulse of 858.3 eV, with a pulse energy of 30 ${J/\text{cm}}^{2}$. Panel (a) shows the total DOS used as an input for the simulation. Panel (b) shows the energy-resolved occupation (between 0 and 1) of the valence band over time, relative to the Fermi energy, and shares the corresponding axes with panels (a) and (c). The population (in electrons/atom/eV) is the product of the DOS and the occupation. The thermalized valence occupation lags a few femtoseconds behind the current chemical potential *μ*; the temperature *T* of the valence system rises rapidly, ultimately reaching up to 25 eV. The bleaching of valence states (highlighted with a blue dotted ellipse) is visible as a high non-thermal population at the resonant photon energy around 7 eV above the Fermi level. Panel (c) shows the number of core holes and free electrons over time, as well as the number of electrons in the valence system below and above the Fermi energy.

Before further interpreting the non-linear effects shown in Fig. 4, let us first consider the example of a local valence band population history as shown in Fig. 5. The example is drawn from the uppermost 4 Å thick voxel of the simulated sample, excited with a Gaussian pulse profile centered around *t* = 0 with 30 fs FWHM duration and 30 J/cm^2^ fluence. As such, the example is selected from the upper range of extreme excitations in this simulation to showcase the effects clearly. While panel (a) of Fig. 4 shows the calculated DOS as used by the simulation and published in Refs. 46 and 47, the colormap in (b) shows the occupation of these states over time. It is apparent that the occupation function mostly resembles a Fermi–Dirac distribution evolving from cold to hot. However, the states at *E_j_* = *E_i_* (highlighted by the blue ellipse), show greater population as they are directly populated by the resonant absorption process. We also show the effective electron temperature *T* and chemical potential *μ*, which are calculated from the internal energy and population of the valence system at every time step. Panel (c) shows the number of electrons per atom in the valence band below and above the Fermi level (blue solid and dashed curves, respectively) as well as the average number of core holes and the number of free electrons over time. One general observation is that for the given 30 fs pulse duration, the number of simultaneously existent core holes remains very small, even for high fluences. This has two reasons: On the one hand, the natural lifetime of the core-holes of 1.4 fs is small compared to the pulse duration.^50^ On the other hand, the monochromatic excitation near the material resonance implies that the photons couple the core-level to a narrow selection of localized valence states.^54^ In this case, the number of resonant valence states is small in comparison to the number of core electrons. Since the core-level and resonant valence states operate like a two-level system in which absorption and stimulated emission compete, the resonant absorption process saturates due to occupied valence states long before the core level is significantly depleted. This bleaching of valence holes is amplified over the pulse duration by an increasingly heated Fermi–Dirac distribution, which also increases the occupation of states above the Fermi level. Since both core-holes and free electrons decay so quickly, a majority of the absorbed energy is quickly translated into a broadening of the valence electron distribution. By the end of the pulse in this example, more than half of the 3d valence electrons are excited to valence states above the Fermi level, while the highest instantaneous number of core holes was only about one per 100 atoms, as shown in Fig. 5(c).

With these general observations about the evolution of the electronic system within the pulse duration in mind, we can now proceed to interpret the mechanisms responsible for the non-linear features in the spectra. Above the absorption edge, the decrease in absorption with increased fluence [see Fig. 4(d)] can be understood as a depletion of valence states available to the resonant core-to-valence transition. Similarly, below the absorption edge, the increase in absorption [see Fig. 4(b)] can be attributed to valence holes below the Fermi-level becoming available due to the thermalization process, as soon as the tail of the Fermi–Dirac distribution reaches the probed energy. The shift of the absorption edge [see Fig. 4(a)] can be explained by a non-linear combination of the two effects above. Consider that below the absorption edge at the beginning of the pulse, the sample only interacts with the x rays via the comparatively weak process of non-resonant absorption. However, once the sample is sufficiently heated that valence holes become available, additional resonant absorption begins to occur and accelerates further electronic heating—and in turn additional pre-edge absorption. Since the onset of this exponential process occurs earlier near the absorption edge, it contributes significantly to the observed spectral redshift. Another cause of the observed edge shift is the shift of the chemical potential *μ*, which strongly depends on the exact shape of the DOS and is shown in Fig. 5(b) as a green line. Initially, *μ* increases with absorbed fluence, as thermally excited electrons from the 3d states must spread out in energy to the lower DOS above the Fermi level. With rising electronic temperature, the high DOS of the 3d states becomes less relevant and the chemical potential drops again as expected in regular metals. A similar evolution of the chemical potential and electronic temperature was predicted for optically excited nickel by previous experiments and calculations.^14,55–57^ It is remarkable that the experimentally observed redshift of 0.9 ± 0.1 eV can be reproduced by the rate model based on this very simple mechanism. However, this mechanism applies specifically to non-linear absorption using monochromatic x rays. Qualitatively similar redshifts have been observed in nickel after excitation with optical lasers even at up to three orders of magnitude lower excitation fluence.^43,58,59^ These redshifts have recently been linked to modifications of the band structure due to the interplay of electronic correlations and optically induced demagnetization.^43^ While such subtle, spin dependent effects may also occur in our high-fluence study, they are evidently overshadowed by the electron population dynamics. This aspect of an initially non-thermal electron distribution evolving toward a Fermi–Dirac distribution was also observed as a critical aspect of the optically excited spectra.^43,60^ The timescale of electron thermalization was estimated to just over 100 fs, which is about 20 times slower than our estimate of 6 fs. This apparent discrepancy results from a scaling of the thermalization time with excitation density. Such a scaling is supported both by theory^52^ and recent pump–probe studies at the nickel M_2,3_ edge^53^ where the electron thermalization time decreased from 34 to 13 fs with rising optical pump fluence from 8 to 62 mJ/cm^2^. This implies that the value of 6 fs found in our study represents an average time constant for the excitation densities in our experiments.

A significant deviation between model and experiment can be observed at the resonance peak itself, where the simulated electron dynamics lead us to expect a much stronger saturation effect than observed experimentally [Fig. 4(c)]. This saturation is reduced as the peak position redshifts and more thermally vacated d-states become resonant, leading the model to predict a slight recovery of absorbance at the highest fluences. Overestimating the saturation effect may be related to a fluence-dependent decrease in the excited state lifetime due to stimulated emission as well as increased carrier mobility around the Fermi edge, both leading to an energetic broadening of the resonant core–valence transition. Such a broadening would increase the number of resonant valence states and thus delay saturation especially at the edge, but is not considered in our model. While it may be expected that a purely population-based model cannot fully represent resonance effects at the resonance peak itself, the lack of any significant saturation around 852 eV [Fig. 4(d)] is more surprising. Both disagreements point to additional physical effects and call for more sophisticated models.

We speculatively propose three mechanisms which could contribute to these discrepancies: First, the transition matrix elements could get modified at higher excitation densities, especially around the resonance, while we model the absorption only based on the ground-state spectrum. Second, an energy dependence of the electron–electron scattering cross section could allow for particularly fast scattering of electrons with certain energies, counteracting the saturation. Third, a collective, correlated response of the electronic system (local field effects) could modify the DOS or the transitions even on the fast timescale of the FEL pulse duration.^43,61^

A more detailed discussion of the model can be found in the Appendixes: In Subsection 4 of Appendix A, we show how a variation or elimination of specific processes leads to different predictions for the spectra, and in Subsection 5 of Appendix A, we discuss the limitations of the rate model and its suitability for future extension.

## VI. CONCLUSION

To summarize, we have analyzed fluence-dependent near-edge x-ray absorption spectra of the nickel 2 $p_{3/2}$ core level up to x-ray fluences of 60 J/cm^2^. We have developed a rate-equation model based on differential equations that describes the excitation and decay processes connecting populations of core and valence electronic states. Process rates are quantified by scaling known ground-state properties with evolving electron populations.

The model enables an understanding of the electronic population history under strong x-ray fluences and characterization of the resulting non-linear absorption near a core resonance. It successfully predicts the observed increase in absorption before and its decrease beyond the resonance, as well as the fluence-dependent redshift of the absorption peak over three orders of magnitude. However, the bleaching of the absorption peak is overestimated by the population-based model and will require more sophisticated models to accurately quantify. Here, the population dynamics rate model also provides a valuable point of reference for more advanced theoretical frameworks.

Providing the fundamental fingerprints of how strong x-ray fluences alter the electronic system and thus the absorption spectra, our straightforward picture of intense core-resonant x-ray pulse interaction can inform the design and interpretation of future FEL experiments. On the one hand, our model can guide the decision up to which point to maximize fluence for good statistics while keeping the absorption process linear, and to recognize the principal spectral fingerprints emerging at the onset of non-linear absorption due to electron dynamics within the pulse. On the other hand, an understanding of the population dynamics within high-fluence pulses, and in particular, an awareness of the dominant influence of electronic scattering processes, is crucial for emerging techniques that aim to utilize x-ray wave-mixing processes, such as stimulated core hole emission, in solids.^40,62–71^

## Figures and Tables

**FIG. 6.**
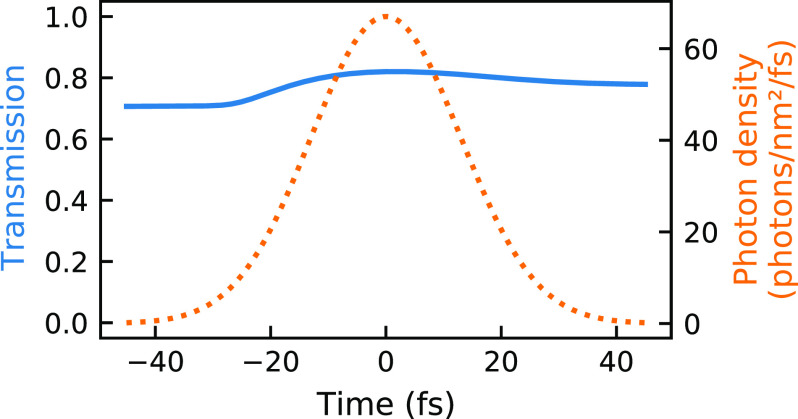
Instantaneous transmission (including resonant and non-resonant absorption) over time for a pulse at 857.5 eV with a pulse energy of 30 ${J/\text{cm}}^{2}$ (blue line, left axis), as well as the temporal profile of the incident photon density (orange dots, right axis).

**FIG. 7.**
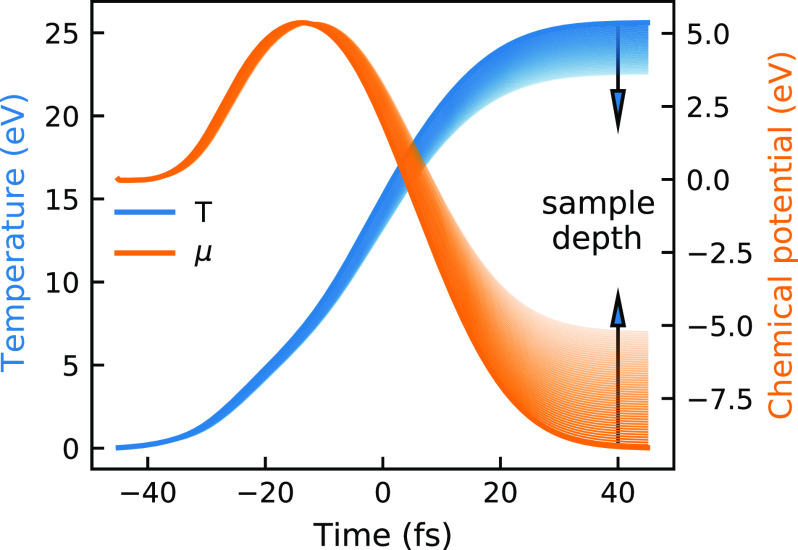
Temperature and chemical potential over time for a pulse at 857.5 eV with a pulse energy of 30 ${J/\text{cm}}^{2}$. The solid line represents the properties at the sample surface and the thin lines represent the deeper layers that are exposed to less x-ray fluence, as indicated by arrows and increasing transparency.

**FIG. 8.**
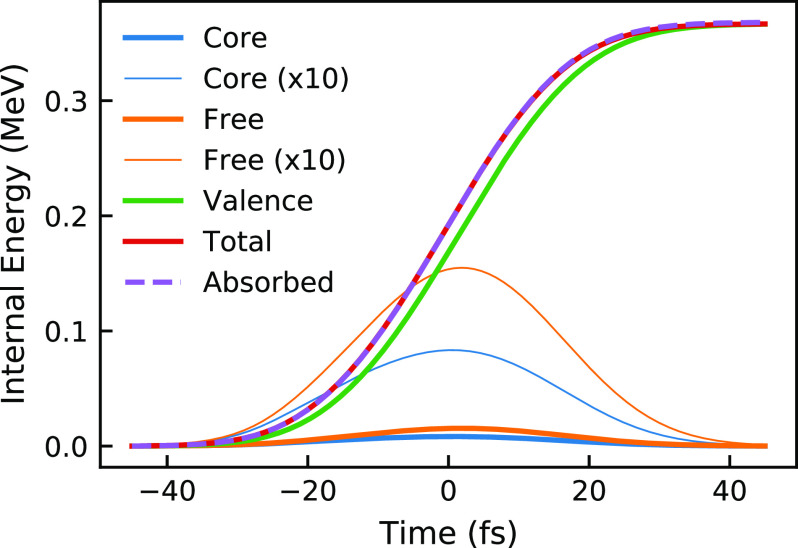
Energy in the sample system over time for a pulse at 857.5 eV, integrated over the full 20 nm thickness of the sample and a 1 nm^2^ area illuminated with a fluence of 30 ${J/\text{cm}}^{2}$. The absorbed energy is calculated from the difference between incident and transmitted photons, while the total energy is a sum of the energy held in the electronic sub-systems of core-holes, free electrons, and valence excitation. Due to the fast process rates in comparison to the pulse duration, the energy held in core excitations and free electrons remains small, which is why a 10 times scaled curve is also shown.

**FIG. 9.**
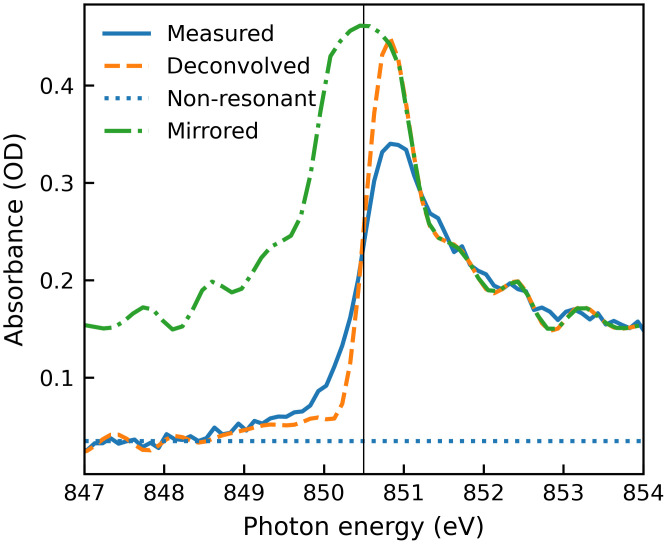
Derivation of absorption lengths as input parameters, reconstructed from a measured ground-state spectrum (blue line). The non-resonant absorption level (blue dots) was determined from the pre-edge region. The measured resonant absorption length was deconvolved with the experimental resolution (orange dashes) and mirrored around the rising edge to retrieve a symmetric resonant absorption length around the resonance (green dotted-dashed line). See main text for details.

**FIG. 10.**
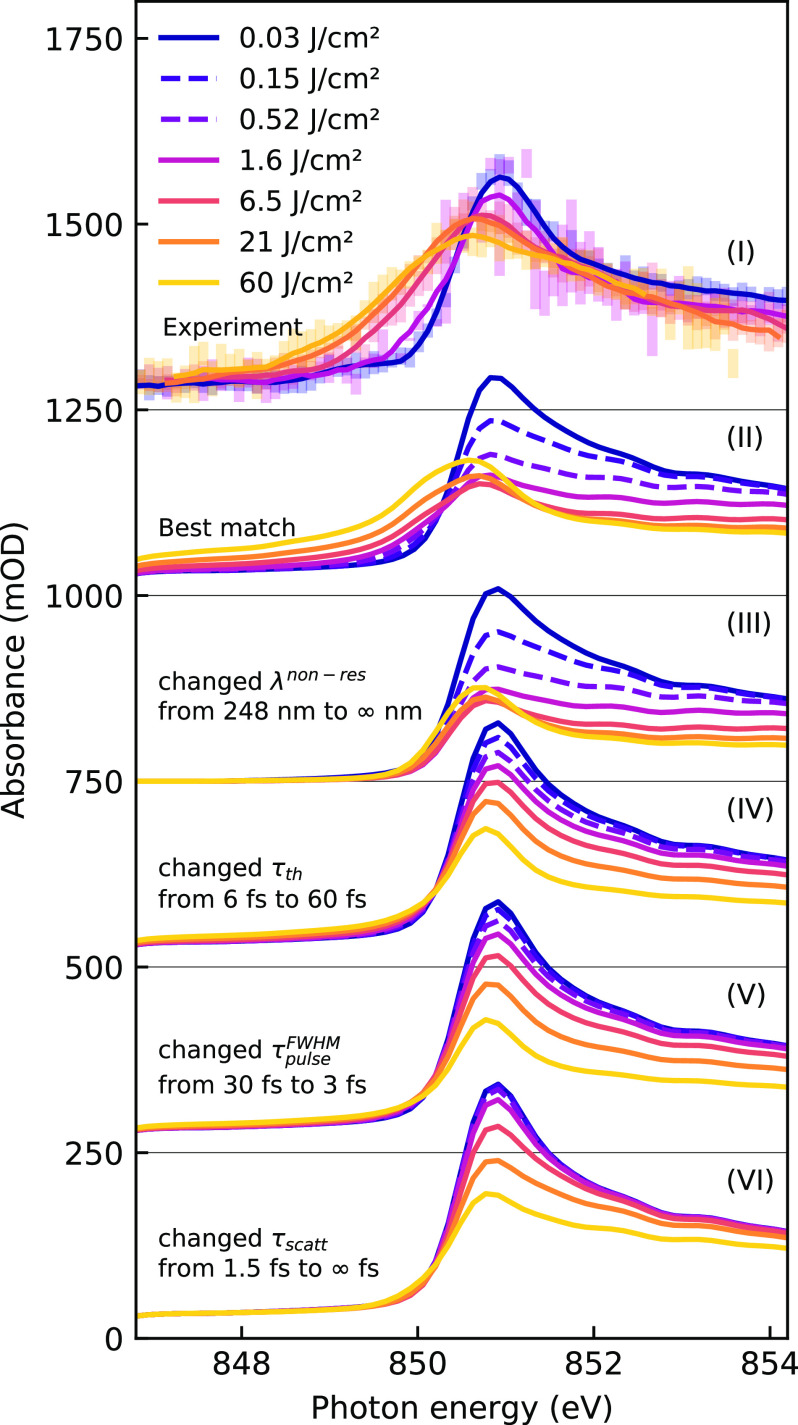
Fluence-dependent Ni $L_{3}$-edge spectra, simulated with different parameters and compared to the measurements. For the spectra (III) to (VI), one parameter was varied with respect to the best match (II). Each set of spectra is offset by another 250 mOD as indicated by the horizontal lines. The error bars of the experimental data represent the 95% confidence intervals for each bin of 102 meV width; the solid lines represent smoothed spectra using a Savitzky–Golay filter using windows of 21 bins and fourth-order polynomials. The average fluence of events contributing to each spectrum is given in the legend. Dashed simulated spectra do not have a corresponding measurement.

**FIG. 11.**
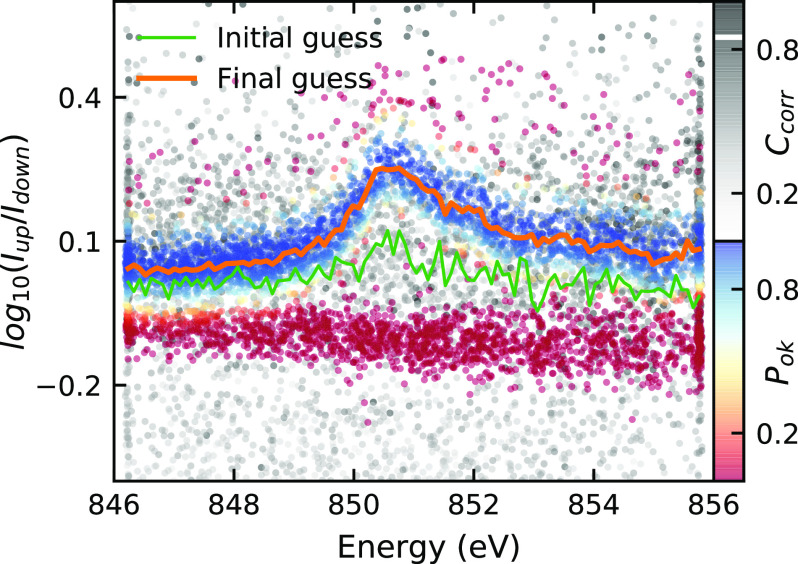
Sorting of FEL shots. Gray points represent events which were excluded based on rigid criteria, mainly their correlation coefficient $C_{\text{corr}}$. Colored points were analyzed using the iterative Gaussian Mixture Model (GMM) optimization. The colorbar shows the estimated posterior probability $P_{\text{ok}}$ that a given shot cleanly probed an unperturbed sample. The green and orange solid lines represent the initial and final average spectrum estimated by the GMM, respectively.

**FIG. 12.**
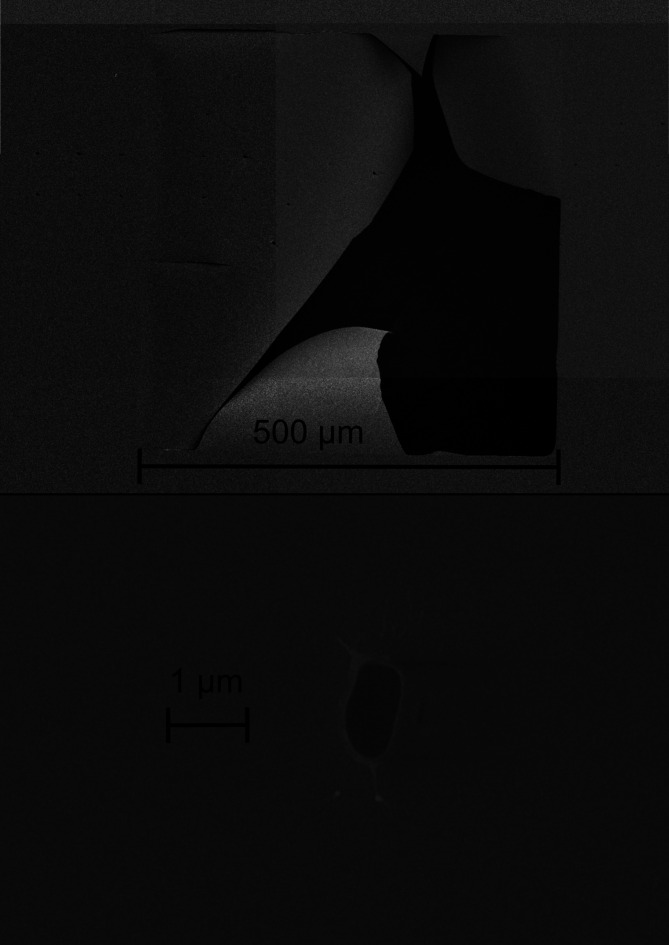
SEM images of the used samples. The top image shows a stitched overview image of a nickel film window. One can see rows of FEL imprints as well as the tearing of the membrane. The bottom image shows a single FEL imprint in a SiN reference membrane.

**FIG. 13.**
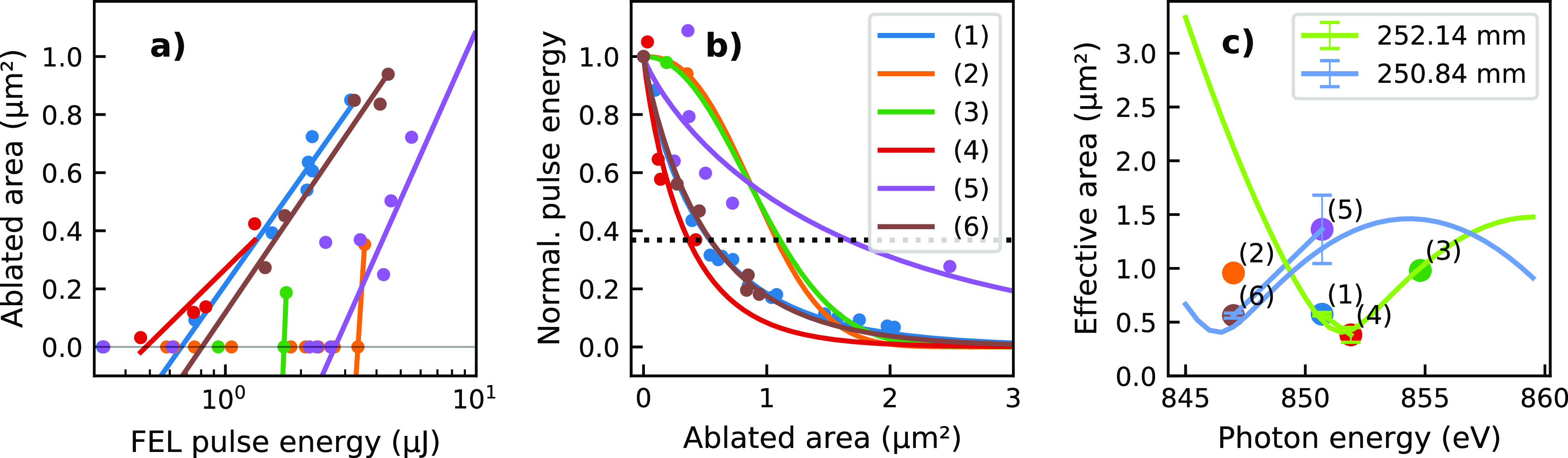
Spot size characterization. (a) Liu's plot to determine the pulse energy damage thresholds. (b) Normalized pulse energy plot to determine the effective area. The legend differentiates the spot groups corresponding to Table IV with colors valid for all three panels. (c) Area of FEL foci, comparing the effective area measurements (dots and error bars) to the ray tracing results (line plots) for two focal distances. For groups (2) and (3) no error estimate could be calculated (see the text).

## Data Availability

Raw data were generated at the European XFEL large scale facility and are available at http://doi.org/10.22003/XFEL.EU-DATA-002170-00. Derived data supporting the findings of this study are available from the corresponding author upon reasonable request. The simulation code that supports the findings of this study is openly available in GitHub, Ref. 72.

## APPENDIX A: THE RATE MODEL

This appendix provides an in-depth discussion of the rate model, including the formalism for each of the modeled processes (Subsection 1 of Appendix A), differentials (Subsection 2 of Appendix A) and the choice of input parameters (Subsection 3 of Appendix A), as well as a study of the models' predictions under different conditions (Subsection 4 of Appendix A). Finally, we present an extended discussion of the applicability and limitations of our rate model (Subsection 5 of Appendix A) beyond the presented dataset.

### 1. Processes

For a better overview, we introduce the processes as individual terms and assemble them into differential equations in Subsection 2 of Appendix A. In principle, each process is described using an absorption length or lifetime which is known from ground-state measurements and then scaled linearly with the changing electron populations with respect to the ground state. The normalization is such that the ground-state rate is reproduced for an undisturbed electron system and the rate vanishes when the corresponding transition cannot happen due to a lack of electrons or holes. We use the indices *i* and *j* to refer to specific energies, where the index *i* is used for photon energies of x rays and the index *j* for the energy of electronic states in the valence band.

#### a. Resonant interaction

The resonant interaction describes both resonant absorption (core–valence transitions) and stimulated emission (valence–core transitions) as a single process. It is calculated for each energy *E_j_* in the valence system that is resonant with a given photon energy *E_i_*,

$$P_{j}^{\text{res}}=\left(\frac{R_{C}}{M_{C}}-\frac{\rho_{j}}{m_{j}}\right)\frac{N_{i}^{\text{phot}}}{\lambda_{i}^{\text{res}}}\delta_{ij},$$
(A1)

where $\lambda_{i}^{\text{res}}$ is the resonant absorption length, *R_C_* is the number of core electrons, *M_C_* is the number of core states, *ρ_j_* is the valence electrons at *E_j_*, *m_j_* is valence states at *E_j_*, $N_{i}^{\text{phot}}$ is the number of photons per ${\text{nm}}^{2}$ at *E_i_*, and *δ_ij_* is Kronecker-delta.

The first terms (in brackets) represent the difference in the occupation of core states $R_{C}/M_{C}$ and resonant valence states $\rho_{j}/m_{j}$. The dominance of absorption over stimulated emission or vice versa is determined solely by this difference, as they represent an optically driven two-level system in the incoherent limit. If the core level population is smaller than the valence population, the resonant interaction process becomes negative, representing the dominance of stimulated emission. The second term on the right is the number of irradiated photons divided by the penetration length. The Kronecker delta ensures that only photons and electrons in corresponding energy bins interact.

#### b. Non-resonant absorption

The non-resonant absorption summarizes photon absorption from other electronic states than the resonant core-level, especially from the valence electrons. Photon densities *N_i_* at all incident energies reduce each population *ρ_j_*,

$$P_{i,j}^{\text{non}-\text{res}}=\frac{\rho_{j}}{R_{V}^{0}}\frac{N_{i}^{\text{phot}}}{\lambda^{\text{non}-\text{res}}},$$
(A2)

where $\lambda^{\text{non}-\text{res}}$ is the non-resonant absorption length and $R_{V}^{0}$ is the total number of valence electrons in the ground state.

The interaction is normalized by the total valence band population in the ground state $R_{V}^{0}$, so that the sum of the first term over all *j* becomes unity if all $\rho_{j}=\rho_{j}^{0}$ (since $R_{V}^{0}\equiv \sum_{j}\rho_{j}^{0}$). The second term represents the non-resonant absorption in the ground state as can be experimentally determined sufficiently before the resonance in the spectrum.

This treatment does not explicitly differentiate the non-resonant absorption from core energy levels other than the one treated by *R_C_*. In the given example with photons resonant to the nickel 2p-absorption, the 3s and 3p core electrons only contribute to a minority of the non-resonant absorption events. In this model, we choose for simplicity to scale this contribution together with the non-resonant scattering from the valence electrons.

An exemplary incidence profile and the resulting transmission over time are shown in Fig. 6.

#### c. Auger decay

The model explicitly treats Auger decay processes that involve one core-hole and two electrons from the valence band. The rate at which an electron in density *ρ_j_* would decay via an Auger process is calculated as

$$P_{j}^{\text{Auger}}=(M_{C}-R_{C})\frac{\rho_{j}}{R_{V}^{0}}\frac{R_{V}}{R_{V}^{0}}\frac{1}{\tau_{C}},$$
(A3)

where *τ*_C_ is the core-hole lifetime and *R_V_* is the total number of valence electrons.

The first factor (in brackets) is the number of unoccupied core states, i.e., core-holes. The second factor describes the relative population of electrons at the energy *E_j_* and the third term is the relative population of the entire valence band to which the electron could transfer its energy. The latter two are normalized by the respective ground-state population. The last term is the decay rate in the ground state, where *τ_C_* represents the ground-state lifetime of a single core-hole. Altogether, this describes Auger decays as interactions between two valence electrons, one emitted, and one filling the 2 $p_{3/2}$ core-hole. In reality, some fraction of Auger decay events will emit electrons from the 3*s* or 3*p* core levels instead, followed by further Auger processes which emit electrons with the remaining energy of the original core-hole. These are not treated separately in our description, since the indirect decay is, on the one hand, a minority contribution and on the other hand, ultimately results in the same energy transfer to the valence band, albeit with a slightly longer time delay due to the intermediate steps.

#### d. Free-electron scattering

Inspired by earlier approaches to a simplified solution of the Boltzmann equation,^73^ we approximate the scattering rates of electrons in terms of characteristic time constants *τ*_scatt_ and *τ*_th_ for the free electrons and valence electrons, respectively.

The lifetime of free electrons *τ*_scatt_ represents the inverse rate at which free electrons *R*_free_ scatter and decay to the valence system. While this parameter is ultimately empirical, it represents a cascade of individual scattering events between electrons. In such a cascade, each free electron eventually transfers all its kinetic energy to the valence system,

$$P^{\text{scatt}}=R_{\text{free}}\frac{1}{\tau_{\text{scatt}}},$$
(A4)

where *τ*_scatt_ is the free electron scattering time constant and *R*_free_ is the number of free electrons.

#### e. Intra-valence redistribution due to scattering

The total rate of electrons redistributed in this time step through scattering *P_red_* is described by two terms. The first term quantifies the total number of electrons that are redistributed in the electron cascades caused by scattering electrons in this time step. The second term distributes this number equally among occupied states *ρ_j_* and unoccupied states *h_j_*,

$$P_{j}^{\text{red}}=\frac{S_{\text{scatt}}-S_{\text{joining}}}{U_{h}-U_{e}}\left(\frac{-\rho_{j}}{R_{V}}+\frac{h_{j}}{H_{V}}\right),$$
(A5)

where *h_j_* is the number of valence holes, $h_{j}=m_{j}-\rho_{j}$; and *H_V_* is the total number of valence holes, $H_{V}=\sum_{j}h_{j}$.

The numerator of the first term represents the total energy that is released by the cascade and is given by the difference between the rates at which energy is released from the free electron energy pool,

$$S_{\text{scatt}}=P^{\text{scatt}}\frac{E_{\text{free}}}{R_{\text{free}}}$$
(A6)

and the rate at which energy is gained in the valence system due to the formerly free electrons occupying random unoccupied valence states,

$$S_{\text{joining}}=\sum_{j}\frac{h_{j}}{H_{V}}P^{\text{scatt}}E_{j}.$$
(A7)

This energy is used to lift a number of electrons from occupied states to higher, unoccupied states. Thus, the denominator represents the energy that the valence system can additionally accommodate, which is the energy that could be contained by filling all valence holes,

$$U_{h}=\frac{\sum_{j}h_{j}E_{j}}{H_{V}},$$
(A8)

minus the energy already contained in the occupied states,

$$U_{e}=\frac{\sum_{j}\rho_{j}E_{j}}{R_{V}}.$$
(A9)

#### f. Electron thermalization

Similarly, *τ_th_* characterizes the time with which the valence system approaches an internal thermal equilibrium,

$$P_{j}^{\text{therm}}=\left[r_{j}(T,\mu )-\rho_{j}\right]\frac{1}{\tau_{th}},$$
(A10)

where *τ_th_* is the valence thermalization time constant, *T* is the equivalent electronic temperature, and *μ* is the chemical potential.

To this end, the chemical potential and equivalent electronic temperature are calculated in each time step based on the current internal energy *U* and number of valence electrons *R_V_*. The Fermi distribution for the calculated chemical potential and temperature then yields a momentary target electron distribution $r_{j}(T,\mu )$, which is approached with the electron thermalization constant *τ_th_*,

$$r_{j}(T,\mu )=m_{j}\frac{1}{e^{(E_{j}-\mu )/k_{B}T}+1},$$
(A11)

$$U=\sum_{j}\rho_{j}E_{j}/\sum_{j}\rho_{j},$$
(A12)

$$R_{V}=\sum_{j}\rho_{j},$$
(A13)

where *U* is the total energy of the valence system, *R_V_* is the current total population of the valence system, $r_{j}(T,\mu )$ is the electron density expected for a fully thermalized valence system, and *k_B_* is the Boltzmann constant.

While the calculation of *r_j_* from *T* and *μ* is straightforward, determining *T* and *μ* from *U* and *R_V_* is an inverse problem. This is solved by iterative optimization using the Levenberg–Marquardt method.^74^

Note that since, by definition, both electron densities—the momentary *ρ_j_* and the thermalized goal distribution *r_j_*—hold the same amount of electrons and internal energy, the sum over the valence band of all electron-distributing thermalization rates is always zero and the change in overall energy is also zero, i.e., $\sum_{j}P_{j}^{\text{therm}}=0$ and $\sum_{j}E_{j}P_{j}^{\text{therm}}=0$.

Figure 7 shows how the temperature (given as an energy $k_{B}T$ in units of eV) and the chemical potential develop over time for a high fluence of 30 ${J/\text{cm}}^{2}$. Although reaching higher temperatures, these results are in qualitative agreement with studies treating the nickel valence system heated with optical lasers.^55,56^

### 2. Differentials

These processes are combined to form the temporal and spatial differentials of the electron, and photon populations *R_C_*, *R*_free_, $R_{V}=\sum_{j}\rho_{j}$, and $N_{i}^{\text{phot}}$. Because the simulation conserves the number of electrons in the sample, in the sum over all *j*, every process term describing electronic transitions appears equally often with positive and negative sign, representing a transition of electrons from one state to another. Only the terms $P_{j}^{\text{therm}}$ and $P_{j}^{\text{red}}$ redistribute electrons exclusively within the valence system without interaction with other pools, appearing only once in the system of equations, but their sum over *j* always equals zero ( $\sum_{j}P_{j}^{\text{therm}}=\sum_{j}P_{j}^{\text{red}}=0$). Each photon energy, *E_i_*, must be resonant to a specific bin of the valence energy system *E_j_*; the Kronecker-delta *δ_ij_* is used to enforce the resonance condition. Note that $N_{i}^{\text{phot}}$ represents flux of a certain number of photons through the sample in each time step. Considering the vacuum speed of light on the given length scale 1/c≃3.3 as/nm, the time the photons require to propagate through the sample is neglected, which greatly reduces computational complexity,

$$\frac{dN_{i}^{\text{phot}}}{dz}\;=-P_{i,j}^{\text{res}}\delta_{i,j}-\sum_{j}P_{i,j}^{\text{non}-\text{res}},$$
(A14)

$$\begin{array}{cc} \frac{d\rho_{j}}{dt} & =P_{i,j}^{\text{res}}\delta_{ij}-\sum_{i}P_{i,j}^{\text{non}-\text{res}}-P_{j}^{\text{Auger}}-\frac{\sum_{j}\rho_{j}P_{j}^{\text{Auger}}}{R_{V}}\\ & +P_{j}^{\text{therm}}+\frac{h_{j}}{H_{V}}P^{\text{scatt}}+P_{j}^{\text{red}}, \end{array}$$
(A15)

$$\frac{dR_{C}}{dt}\;=-\sum_{i,j}P_{i,j}^{\text{res}}\delta_{i,j}+\sum_{j}P_{j}^{\text{Auger}},$$
(A16)

$$\frac{dR_{\text{free}}}{dt}\;=\sum_{i,j}P_{i,j}^{\text{non}-\text{res}}+\sum_{j}P_{j}^{\text{Auger}}-P^{\text{scatt}}.$$
(A17)

#### a. Free electron energy

While the particle number involved in the simulation is naturally enforced by the symmetric use of each process term in the differentials (see Sec. III A of the main text), the energy within the system is also a conserved quantity. Since the core-level is considered energetically sharp and the valence-system is modeled energy-resolved, the internal energy of the system can be calculated as a simple sum over the product of energy levels and their current populations. Only for the pool of free electrons *R_free_*, the energy-distribution of the contributing electrons is not explicitly tracked. Instead, we track the total energy stored in this pool with the parameter *E_free_* using the following additional differential:

$$\begin{array}{cc} \frac{dE_{\text{free}}}{dt} & =\sum_{i,j}P_{i,j}^{\text{non}-\text{res}}(E_{F}+E_{i}-E_{j})\\ & +\left(\frac{\sum_{j}E_{j}(\rho_{j}-P_{j}^{\text{Auger}})}{\sum_{j}\left(\rho_{j}-P_{j}^{\text{Auger}}\right)}+E_{F}\right)\sum_{j}P_{j}^{\text{Auger}}\\ & -P^{\text{scatt}}\frac{E_{\text{free}}}{R_{\text{free}}}, \end{array}$$
(A18)

where *E_F_* is energy of the Fermi level.

Because photons can “kick out” electrons from arbitrary states in the valence system, it becomes necessary to track the total kinetic energy of the free electrons, even though the distribution of energy among these electrons is not tracked. The rate of energy transfer to the free electron bath is described as the sum of non-resonant absorption and Auger decay processes, each multiplied by their respective energies (first and second line, respectively). The energy of the secondary Auger electrons is calculated as the average energy of all electrons other than the primary Auger electrons, as those drop to the core level. Finally, each electron that leaves *R*_free_ reduces the energy of the bath by the average energy, which is $\frac{E_{\text{free}}}{R_{\text{free}}}$.

Explicitly tracking this energy also enables us to demonstrate the conservation of energy within the simulation; since there is no channel that allows energy to leave the sample, the energy held in the electronic sub-systems matches that of the absorbed photons at all times. Figure 8 shows the internal energy of the electronic subsystems over time, integrated over an area of 1 nm^2^ and the full 20 nm thickness of the sample. The comparison of the energy in various sub-systems demonstrates how quickly the energy of the photon pulse is transferred to valence excitations. Furthermore, observing the energy conservation has proven to be an invaluable tool to select sufficiently fine binning in time, space and energy, as it is particularly sensitive to the accumulation of numerical errors. For example, the calculation for the homogeneous illumination with 30 ${J/\text{cm}}^{2}$ shows a cumulative error in energy of 0.35%. This is in contrast to the electron conservation, which is strictly kept to machine precision level due to the symmetric way the process terms are arranged to form the time differentials.

### 3. Parameters

There are four categories of parameters: First, resolution parameters for the number of steps in time, space, and energy are chosen as a compromise between calculation time and numerical error; second, experimental parameters such as the pulse duration, peak fluence, and bandwidth of the interacting photons reflect experimental conditions; third, ground-state properties such as the atomic density and number of states and electrons, as well as Auger-decay limited core-hole lifetime are drawn from published literature, while the resonant and non-resonant absorption lengths are derived from the ground-state spectrum as described below. Fourth, the model-inherent phenomenological parameters are the valence thermalization time *τ*_th_ and electron scattering time *τ*_scatt_, which are varied to achieve the best match to the experimental results. A list of the relevant parameters and the chosen values for the present calculations is shown in Table III.

**TABLE III.** Parameters for the presented simulation results. The first block lists parameters that define the resolution of the simulation, the second block shows experimental conditions, the third phenomenological fitting parameters and the last block contains physical ground-state properties of the sample.

Symbol	Code	Description	Unit	Value
*N_z_*	Nsteps_z	Steps in sample depth	⋯	50
$N_{E_{j}}$	N_j	Steps in energies considered in valence system	⋯	90
$N_{E_{i}}$	N_points_E	Number of photon energies/points in the spectrum	⋯	69
⋯	N_local_fluences_to_calculate	Number of fixed fluences that are directly simulated	⋯	30
⋯	N_pulse_energies	Number of final pulse energies with a Gaussian spot profile	⋯	20
⋯	Nsteps_r	Number of steps in the radial integration of the Gaussian spot	⋯	100
*dt_min_*	timestep_min	Minimum allowed time step	fs	0.15
⋯	Energy_axis_max	Maximum energy in the valence system	eV	800
⋯	Energy_axis_fine_until	Finer sampling for energies lower than this	eV	30
⋯	Energy_axis_min	Valence band origin	eV	−10
⋯	DOS_band_origin	Energy minimum from where to use the loaded DOS	eV	−10
⋯	DOS_band_dd_end	Energy maximum from where to use the loaded DOS	eV	30
*σ_tj_*	tdur_sig	Rms pulse duration of photons	fs	13
*E_i_*	E_i	Photon energy of incident photons	eV	848–856
*I*_0_	I_0	Total number of photons in the simulated pulse	photons nm^−2^	$0-1.4\times 10^{4}$
*T*_0_	temperature	Initial sample temperature	K	300
*σ*_BW_	interaction_bandwidth	Bandwidth of resonant interaction at *E_j_*	eV	0.638
*τ*_scatt_	tau_scattering	Scattering time of free electrons	fs	1.5
*τ*_th_	tau_th	Thermalization time of non-thermal valence states	fs	6
-	DOS_shapefile	Filename of the total DOS from DFT-calculation	⋯	from Ref. 46
*Z*	Z	Total sample thickness	nm	20
*ρ*	atomic_density	Atomic density	atoms nm^−3^	91.4
$R_{V}^{0}$	valence_GS_occupation	Valence electrons per atom in the ground state	states atom^−1^	10
*M_C_*	core_states	Core electrons/states per atom in the L_3_ core level	states atom^−1^	4
*E_f_*	E_f	Fermi level; used as zero for energy axes of *E_j_* and *E_i_*	eV	850.5
*τ_C_*	tau_core_hole	core-hole lifetime, from Ref. 50	fs	1.4
$\lambda^{\text{non}-\text{res}}$	lambd_nonres	Absorption length due to non-resonant absorption	nm	248
$\lambda_{Ej}^{\text{res}}$	lambd_res_Ej	Absorption length due to resonant absorption	nm	20–83

The parameterization of several ground-state properties deserves further comment. First, the DOS (motivated at the start of this section) was subdivided into bins *m_j_* of varying size, favoring a fine resolution for the bound states.

The size of the energy bin that is resonantly coupled to the core level by the incident photons is chosen such that it represents the interaction bandwidth of the photons. The interaction bandwidth is calculated as the convolution of the bandwidth of incident photons (i.e., the resolution of the experiment) and the natural linewidth of the core excitation. We further account for the final state broadening of excitations into less tightly bound states by enlarging the interaction bandwidth by 0.1 eV per eV above the Fermi level. Furthermore, the resonant and non-resonant absorption lengths are derived from the ground-state spectrum. We treat the non-resonant absorption length as constant, i.e., independent of photon energy, and derive it from the pre-edge absorption level. The transition matrix element of a core–valence transition exhibits a resonant enhancement close to the absorption edge, which translates into an energy dependence of the resonant absorption length. Above the Fermi level, where the DOS is unoccupied in the ground state, the resonant absorption length is encoded in the ground-state absorption spectrum. As the transition matrix element from the core level to states below the Fermi level is experimentally not straightforward to access, we use the approximation that the energy-dependence of the transition matrix element is symmetric around the Fermi level. To derive the resonant absorption length, the non-resonant absorption level is subtracted from the spectrum and line broadening is accounted for by deconvolution with a pseudo-Voigt-profile of 50% Gaussian and Lorentzian share and a width of 640 meV FWHM, representing 420 meV broadening from the experimental resolution and 480 meV from the core-hole lifetime.^50^ The deconvolved resonant absorption spectrum above the Fermi level is then mirrored around the Fermi level and the discontinuity within 320 meV around it is reconstructed with cubic interpolation. This results in the mirrored resonant absorption spectrum shown in Fig. 9, which is used as the resonant absorption length parameter $\lambda_{i}^{\text{res}}$. Note that the results of simulated spectra were finally re-convolved with the same pseudo-Voigt-profile to simulate the same experiment.

### 4. Rate model parameter study

In Fig. 10, we show the measured non-linear x-ray absorption spectra labeled (I) together with sets of simulated spectra computed for different sets of parameters.

The first set of simulated spectra represents the best match with the experimental conditions with the parameters shown in Table III and is labeled (II), while the consecutive sets, labeled (III) to (VI), demonstrate how the results change when individual parameters are modified. We present this set of simulations to show how our model may be used to understand the relation between the non-linear changes and various parameters. To fit the experimental results, however, only the parameters *τ_th_* and *τ*_scatt_ are treated as unknowns, while all other parameters are known experimental or ground-state parameters.

In the best matching simulation (II), the experimental observations of a redshifted rising edge, increased pre-edge absorption, as well as reduced absorption at and beyond the absorption peak, are reproduced. However, the saturation effect at the resonance is over-estimated and the lack of measured saturation around 852 eV cannot be explained by our model.

The next simulation (III) was performed without non-resonant absorption. While this eliminates the pre-edge absorption rise, some shift of the absorption onset is still predicted within the original peak, while the spectra above the resonance onset behave similarly to the best-matching simulation.

With simulations (IV) and (V), we demonstrate the effect of prolonging the thermalization time or shortening the pulse duration by a factor of ten, respectively. Both have the similar effect of strongly reducing the peak shift and a moderate decrease in saturation beyond the resonance.

Finally, we show non-linear spectra (VI) where the free electron scattering process was eliminated ( $\tau_{\text{scatt}}=\infty$). This prevents the majority of the excitation energy from entering the valence system and thus drastically reduces valence heating. This causes the pre-edge absorption rise and rising edge shift to vanish nearly entirely, and absorption-decrease due to saturation at and above the resonance is also strongly reduced. These differences underscore the importance of electronic scattering cascades to these phenomena.

While an extensive study of the correlations between the various model parameters and the observed effects is beyond the scope of this work, this brief parameter study allows an interpretation of how redshift and pre-edge absorption rise occur: Let us consider a case where the incident photon energy is slightly below the absorption edge. Initially, only non-resonant absorption transfers energy to the sample, specifically by creating free electrons in form of photo-electrons. This energy is then transferred to the valence band due to electronic scattering cascades, where it causes many small, non-thermal excitations. These are homogeneously distributed over all valence energies and, thus, only lead to a small increase in pre-edge absorption (see simulation IV with slow thermalization). However, this distribution of excitations develops toward the shape of a Fermi distribution over time, specifically with the thermalization time constant. If the pulses are long in comparison to the thermalization time, a significant number of states below the Fermi level becomes available for core–valence transitions during the pulse duration. This causes the observed increase in pre-edge absorption. Once the first empty valence states become available at the current photon energy, resonant absorption begins to occur in addition to the non-resonant absorption. The additional resonant absorption leads to more free electrons from Auger-decay, which in the same way as the photo-electrons further contribute to secondary electron scattering and thermalization. Because thermalization mostly creates free states just below the Fermi level, this process of self-enhancing rise of overall absorption manifests in a fluence-dependent shift of the rising edge to lower energies.

### 5. Limitations and potential

As demonstrated in the parameter study above, this kind of analysis can provide a straightforward interpretation of how non-linear changes to the absorption spectrum emerge, and what each change says about the development of the electronic populations. While the model already demonstrates good agreement with experimental results, below we discuss the limitations of our approach and possibilities for expanding or refining it.

One particular physical effect that has been observed in warm dense matter is ionization potential depression. Here, the absorption edge is lowered for those atoms which have already been ionized, and manifests typically in discrete peaks emerging before the edge, see, e.g., Ref. 75. This signature is not visible in the spectra, likely because the non-resonant absorption at the photon energies where this could have been observed was not strong enough to ionize a significant number of atoms. It was, therefore, also omitted in the simulations. It may, however, be relevant when simulating spectra measured with higher fluence, a broader bandwidth or two pulses with separate photon energies (e.g., below and above the edge). Including it in the simulation could be possible by adding additional resonant absorption channels with a shifted core level, scaling their rate proportional to the fraction of ionized atoms.

In the model as described here, the DOS is not only assumed constant but also does not differentiate between spin-up and spin-down states, although we are treating a magnetic material. Splitting up the DOS in spin-up and spin-down states would allow for the inclusion of angular momentum conservation and transfer in the various scattering rates.

While phonon-mediated energy transfer is usually negligible in the first 100 fs after excitations, spacial coupling due to ballistic electrons can become relevant if the electron mean free path becomes similar to the sample thickness or the absorption length.^76,77^ In this case, the model assumption of strictly local electronic excitations would have to be carefully reevaluated. While an additional process for ballistic electrons leaving the sample or interacting across the interface with a substrate could be introduced, directly calculating spatial coupling of electronic excitations within the sample in three dimensions would dramatically increase the computational complexity.

Fluorescent decay should be accounted for when moving to harder x rays.^49^ While introducing the decay channel itself would be simple, accurately accounting for reabsorption may be less straightforward, since the model only propagates light in one direction.

Furthermore, the thermalization time *τ_th_* is used in this work as a global fitting parameter, although electron thermalization times have been suggested to depend on electronic temperature.^52,53^ Since the electronic temperature and target distribution are calculated every time step, an arbitrary dependence could be easily introduced, albeit with the necessity of additional fitting parameters or predetermined knowledge about the scaling of electronic temperature.

The DOS is dominated by the crystal lattice, which is typically stable on the sub-100 fs timescale. However, recent (time-dependent) density functional theory (TD)DFT calculations show that electronic processes causing sub 100 fs demagnetization via spin–orbit coupling can also lead to modifications of the DOS due to the presence of local electronic correlations modeled by introducing an onsite Hubbard correlation *U* to the mean-field Hamiltonian.^43^ Since the rate model approach is generally not suited to calculate the DOS, incorporating modifications to the DOS would require a close interplay of the rate model with (TD)DFT calculations, leading to an ultimately much more complex approach reminiscent of models developed for the study of radiation-induced damage mechanisms.^23,24^

Furthermore, we derive the interaction bandwidth (the valence energy range to which core states can be resonantly coupled by incident photons) as a convolution of instrumental resolution and the lifetime of the core excitation, i.e., the Auger lifetime. The final state lifetime broadening of excitations into continuum states is described as a continuous broadening of 0.1 eV per eV above the Fermi level.^78^ It is however reasonable to expect that the final state lifetime is further shortened at higher fluences, due to increased rates of both electronic scattering and stimulated emission; the latter would be particularly relevant at the resonance peak. Such further broadening would cause more valence states to be available for resonant interaction and reduce the observed saturation effect while increasing the number of core-holes that may be created by high fluences.^79^ Taking into account for a fluence-dependent broadening of the interaction bandwidth in a refined rate model might help to remedy the overestimation of the saturation effect at the absorption peak in the presented calculations.

Another candidate for further refinement is an energy dependence of the electronic scattering rates. The presented model uses fixed rates for thermalization and scattering cascades, which both act on all valence states indiscriminately. This description would be especially inadequate when applied without modifications to a bandgap material. An advanced model could describe both the thermalization rate of the valence band and the energy of excitations from scattering cascades in an energy-resolved manner.^80^

While such refinements may seem attractive, a core strength of the rate model approach is its relative simplicity and computational tractability, as well as the use of known ground-state parameters, which supports a straightforward physical interpretation. It is ultimately a mostly classical, phenomenological model which offers a complementary approach to *ab initio* calculations. Every added complexity should therefore be weighed against its relevance, as a simpler model facilitates a meaningful interpretation and avoids introducing ambiguity in the results due to correlations between redundant input parameters.

Since the model operates on widely applicable principles, we expect that it may be applied to a wide range of materials with some predictive power, while the limitations described above apply. The results will especially deviate from observations wherever multi-particle effects, such as electron correlations or quasiparticles become relevant.

## APPENDIX B: EXTENDED INFORMATION ON THE EXPERIMENT

This appendix provides details on the experiment, namely, about the procedure of the data acquisition (Subsection 1 of Appendix B), the calibration of the main detector (Subsection 2 of Appendix B), the classification of individual FEL shots (Subsection 3 of Appendix B) and finally on the determination of the effective FEL spot size (Subsection 4 of Appendix B).

### 1. Data acquisition

The experimental x-ray absorption spectra presented here were collected in the scope of a community proposal as the first user-beamtime at the SCS instrument. The intensities of the two beams generated by the beam-splitting zone plate were recorded using a FastCCD detector^34^ with 1920 × 960 pixels of 30 × 30 $\mu m^{2}$. The intensities of both beams were integrated over a region of interest (ROI) corresponding to 350 × 350 pixels each to retrieve the signal and reference intensities for each FEL shot. The high beam divergence due to the zone plate focusing distributed the signal on a 4 mm wide square on the detector 1 m downstream of the sample, thus greatly decreasing the fluence incident per detector area in order to avoid detector saturation. The gas-attenuator was filled with varying low pressure of nitrogen gas to regulate the transmission through the beamline to the required fluence. We refer to low-intensity spectra if the fluence was consistently below the sample damage threshold and the full measurement could be recorded on a single spot, without scanning the sample.

For measuring high-intensity spectra, the fluence often exceeded the material damage threshold, creating shot craters and sometimes causing larger fractures in the support membrane. For measurements at these fluences, the sample holder was scanned at a speed of 0.5 $\text{mm}\;\;s^{-1}$. Therefore, only about 50% of all FEL shots were transmitted through the windows; in the other cases, one or both beams were blocked or clipped by the frame. The membranes were arranged on the frame in a periodic pattern of two rows of sample and two rows of reference membranes with a distance of 1 mm between rows. This ensured that the two FEL foci always impinged on one sample and one reference membrane; a third row was unused in between. Therefore, every time the currently scanned rows were incremented, the upper beam would switch from probing reference membranes to probing sample membranes or vice versa, while the opposite holds for the lower beam. To prevent detector saturation, an additional aluminum filter of about 13 *μ*m thickness was installed in front of the detector during these measurements.

### 2. Detector calibration

The temperature of the FastCCD rose consistently during operation and required cooldown periods in between measurements, leading to the temperature varying between −27 and −5 °C not only over time during the measurement, but also spatially over the detector area. The detector dark signal, as well as the gain coefficients for the three gain settings between which the detector pixels switch automatically, depend on the detector temperature. This made it necessary to reconstruct a temperature-dependent gain calibration. The three temperature-dependent background levels were drawn from dark images collected at various temperatures for each gain setting; the gain coefficients for each setting were drawn from a statistical analysis of the observed gain switching thresholds, such that the calibrated histogram of pixel intensities becomes continuous over all three gain levels. While the calibration accounts for the temperature measured using a temperature sensor on the detector, spatial variations over the detector area remain. The primary effect of this temperature variation was a varying background signal, following a spatial exponential distribution between the detector center and rim, with a higher baseline near the detector center. To account for this, an estimated background signal was derived from the measurements themselves: For a running average of 100 images, the illuminated area was cut out and interpolated using fits to the background level in the non-illuminated area. This additional background variation was then integrated into the gain calibration described above. Furthermore, a mask of hot and dark pixels with irregular behavior was generated from separate measurements and the respective pixels were excluded from the analysis. Despite these corrections, the detector inhomogeneities constitute a significant part of measurement uncertainty in the presented spectra. In particular, the uneven warming of the two detector halves on which the upper and lower beam impinged has the potential of introducing systematic uncertainties. Thus, the shot-sorting algorithm described below was applied separately for all rows where the sample was in the upper beam and the reference in the lower and then to all rows where the orientation was reversed. Fortunately, differences between the temperature of both detector hemispheres affect the two equally sized groups of data (sample up and sample down) with equal and opposite magnitude. Therefore, possible systematic deviations are eliminated in the average over both groups and instead contribute to the statistical uncertainty which is represented in the error shown in Fig. 3 of the paper.

It is useful to mention to potential future users of the beamline that the difficulties with hardware calibration and data pipeline described above were a feature of this first user experiment and have since been addressed. The evolved version of the here described setup is now in routine operation and has since been shown to operate with a sensitivity close to the photon shot-noise limit.^32^

### 3. Event classification

Furthermore, whenever the sample or reference membrane was torn due to a particularly intense shot, subsequent shots sometimes impinged on the torn rim of the sample, possibly at an angle to the membrane surface, or did not hit any sample material at all. Shots affected in this way were not always trivial to identify from any single measurement parameter, which lead to the following procedure to identify and exclude faulty FEL shots: First, the detector image in the ROI around one beam was compared to an extended ROI around the other beam using a normalized two-dimensional cross correlation algorithm. For this, the images were first smoothed by convolution with a Gaussian kernel to remove the influence of the rough surface structure of the aluminum filter before applying the cross correlation function, both algorithms implemented in the scikit-image^81^ package. This procedure yields a correlation coefficient and a displacement vector. The correlation coefficient was used as an indicator that both beams were transmitted through a window without significant differences in the wavefront.

Since data acquisition of the motor encoder for the position transverse to the scanning direction was not working, the real path of the beam over the sample frame was reconstructed by combining knowledge of the beam position along the scanning direction and the manual notes in the laboratory book with the correlation coefficients between both beams on the detector. In the later analysis of the damaged samples, this allowed associating specific shot craters scrutinized with SEM to specific FEL shots.

From the FEL shots which hit sample windows according to this reconstruction, outliers were dropped if either the correlation coefficient dropped below 85% or the displacement vector deviated by a significant margin (manually calibrated for each measurement setting) from the expected beam splitting. Likewise, extreme outliers in the ratio of reference to sample intensity were also dropped at this stage. These criteria proved to be largely redundant as they mostly agree with each other on which events to exclude. Using all of these criteria, events where one or both beams are blocked or clipped can be excluded reliably. In Fig. 11, these excluded points are shown in gray. Each dot represents one FEL shot; its y-position is the logarithmic ratio between sample and reference intensity; the x-position is the photon energy setting of the monochromator, while the gray-scale encodes the Pearson correlation coefficient $C_{\text{corr}}$ of the two regions of interest.

From the distribution of the remaining events, shown in color, it is obvious that further classification is needed. This is because shots onto a damaged or missing membrane can produce spots with good correlation and the expected displacement vector. These can only be distinguished by the fact that the apparent optical density deviates unreasonably from the expected value at the given photon energy. However, this transmission ratio is also the quantity of interest for the final spectrum. To disentangle events affected by prior sample damage from true measurements, an iterative approach using a Bayesian Gaussian mixture model was utilized: To start, an initial guess for a spectrum is computed using the events that passed filter conditions described above and shown as a green line in Fig. 11. Then, for each FEL shot, the deviation of the logarithmic ratio between sample and reference intensity from the initially guessed spectrum is computed. This allows for generating a histogram of these absorbance deviations from the initial guess. Assuming a good guess of the initial spectrum, one may expect that the “good” FEL shots are normally distributed around zero deviation, while shots affected by various sources of uncertainty are distributed with some other distribution, dependent on the type of uncertainty. Thus, the histogram is fitted with four Gaussian distributions which are used as prior probability distributions, the first of which corresponds to good FEL shots. Then, the posterior probability of belonging to the category of good shots is computed for each FEL shot. These posterior probabilities are used as statistical weights to compute an improved guess of the measured spectrum. This improved guess was convoluted with a Gaussian kernel to prevent over-fitting before using it as a new initial guess for the Gaussian mixture model. The spectrum is considered converged when the average change per iteration anywhere in the spectrum is less than 5 μOD. This procedure converges (if at all) within a few (between 3 and 20) iterations to a solution that is robust even against a strong variation of the initial guess. In Fig. 11, the shots analyzed in this way are shown in colors encoding the final posterior probability $P_{\text{ok}}$, indicating the estimate of the model about the validity of each shot. The final guess of the average spectrum is shown as an orange line.

This final procedure rejects outliers purely based on their deviation from the expected value, which requires further justification: In this case, the procedure should return valid results under the condition that all valid FEL shots at a certain photon energy measure a transmission value within a single continuous range. The width of the distribution is resulting from a combination of non-linear changes and measurement noise. This condition must be fulfilled in the present case as long as the fluence-dependent transmission curve of the sample is continuous and a continuous range of pulse energies is contained in the dataset (which is the case for SASE fluctuations). Apart from this logic, the rejected outliers do not appear systematic, further supporting the applicability of the algorithm.

### 4. Determination of the effective spot size

The zone plate has a size of ${\left(1\;\text{mm}\right)}^{2}$ and combines a focusing Fresnel zone plate, off-axis by 0.55 mm with 250 mm focal length (at 860 eV) with a line grating with 379.4 nm pitch in a single optical element. Details on the zone plate can be found in Ref. 29. While the monochromator was scanned between 846 and 856 eV, the effective size of the foci on the sample changed due to the wavelength dependency of the zone plate diffraction. The photon energy-dependent focal size of the zone plate foci was calculated by ray optics calculations based on the beamline settings.^82^ Uncertainties in the exact beam path parameters along the beamline were accounted for by matching the ray optics calculations to effective spot size estimates derived from analyzing the shot craters on the used samples using a simplified form of the procedure laid out in Refs. 83 and 84. Since a full intensity profile could not be measured from the shot craters, the concept of the effective area of the focal size is used. This area connects the peak fluence *F*_0_ with the overall pulse energy $E_{\text{pulse}}$, i.e.,

$$A_{\text{eff}}=\frac{E_{\text{pulse}}}{F_{0}},$$
(B1)

and it can be defined for an arbitrary spot profile.

To characterize the effective focal size, the reference membranes were analyzed using SEM. Figure 12 shows two SEM images: one overview image of an entire membrane and one example of a high-resolution image of a single imprint. Such high-resolution images were taken of 85 selected spots which were associated with the corresponding FEL photon diagnostics data by matching the reconstructed movement of the sample stage to the pattern of imprints on the sample. The reference membranes were chosen for the imprint analysis since their x-ray absorbance can be considered constant for the scanned photon energies.

Since the zone plate is a diffractive element, its properties such as focal length are energy-dependent. Furthermore, the fluence-dependent spectra presented in this paper are combined from measurements at two distinct object distances of 250.84 and 252.14 mm.

Thus, the SEM images were grouped into six groups (see Table IV) by focal length and photon energy of the associated shot, and the total damaged or ablated surface area was determined for each shot. For each group, the minimum FEL pulse energy at which damage is observed on the reference membranes was determined by a Liu's plot^85^ as shown in Fig. 13: The ablated area was plotted over the logarithm of the shot energy and a linear fit was applied to the shots with less than 1 $\mu m^{2}$ ablated area to determine the pulse energy damage threshold.

**TABLE IV.** Results of the spot size characterization. The object distance refers to the distance between sample and zone plate. Pulse energies are shown as measured at the XGM without accounting for the efficiency of the zone plate (about 9%) and the transmission of the beamline KB-mirrors (about 80%). The effective area of groups 2 and 3 is under-determined (see the text).

Group	Object distance (mm)	Photon energy (eV)	Pulse energy threshold ( μJ)	Effective area ( $\mu m^{2}$)
1	252.14	850.7	0.308	0.584
2	252.14	847.0	3.374	0.957
3	252.14	854.8	1.712	0.981
4	252.14	851.9	0.482	0.383
5	250.84	850.7	2.733	1.618
6	250.84	847.0	0.807	0.567

Following the concept outlined in Ref. 83, the pulse energy of all shots was then normalized using this damage threshold to derive the normalized fluence level *f*(*S*) as a function of the ablated area *S*. If the spot size were Gaussian, the function *f*(*S*) should be a simple negative exponential, and the effective area would correspond to the value of *S* where *f*(*S*) equals 1/e, which is indicated in Fig. 13(b) as a horizontal dotted line. For the given non-Gaussian case, the function *f*(*S*) was fitted with a modified exponential $f(S)=e^{\left(-aS^{b}\right)}$, yielding for each group of shots fit parameters *a* and *b* and their uncertainties *σ_a_* and *σ_b_*. The effective area is then calculated as the integral of *f*(*S*). For the estimate of the uncertainty shown as error bars in Fig. 13(c), the integral was also evaluated for $a+\sigma_{a}$ and $b+\sigma_{b}$ as well as $a-\sigma_{a}$ and $b-\sigma_{b}$. Groups (2) and (3) each contained only a single FEL shot with visible damage. While this does allow for an estimate of the damage threshold [compare Fig. 13(a)], the function *f*(*S*) shown in (b) is barely determined by fitting only to the normalization point and a single further point. Thus, no mathematical uncertainty estimate can be given and the resulting points (2) and (3) shown without error bars in Fig. 13(c) should be considered as tentative estimates.

The ray optics calculation tracks the beams in the vertical and horizontal planes. The focus area was calculated from the ray optics calculations assuming an elliptical beam shape. It revealed a slightly astigmatic focus on the sample, due to the beamline optics using separate horizontal and vertical focusing, thus illuminating the zone plate with slightly non-uniform beam divergence. The separate location of horizontal and vertical foci leads to the two minima visible in Fig. 13. Based on the measurements, a minimal beam waist radius of 80 nm was imposed on the ray tracing calculations, accounting for imperfections in beam quality and optics. Normalizing the beam damage thresholds by the thus determined effective area, we find on average that the reference membranes where damaged above fluences of about 10.5 J/cm^2^.

The fluences for the high-fluence spectra shown in this paper are calculated based on the pulse energy measured by the XGM behind the monochromator, multiplied with the efficiency of the focusing optics (80%)^86^ and zone plate (9%) and divided by the effective area derived with the presented ray optics calculations. Since the XGM was calibrated for high pulse energies, the fluences for the low-fluence spectra are based on the reference intensities measured on the CCD and calibrated to be consistent with the reference intensities measured for the high-fluence data, accounting for the calculated transmission of the aluminum filter.
